# Important ecophysiological roles of non-dominant *Actinobacteria* in plant residue decomposition, especially in less fertile soils

**DOI:** 10.1186/s40168-021-01032-x

**Published:** 2021-04-07

**Authors:** Yuanyuan Bao, Jan Dolfing, Zhiying Guo, Ruirui Chen, Meng Wu, Zhongpei Li, Xiangui Lin, Youzhi Feng

**Affiliations:** 1 State Key Laboratory of Soil and Sustainable Agriculture, Institute of Soil Science, Chinese Academy of Sciences, Nanjing, 210008 People’s Republic of China; 2 University of Chinese Academy of Sciences, Beijing, 100049 People’s Republic of China; 3 Faculty of Engineering and Environment, Northumbria University, Newcastle upon Tyne, UK; 4 Soil Subcenter of Chinese Ecological Research Network, Institute of Soil Science, Chinese Academy of Sciences, Nanjing, 210008 People’s Republic of China

**Keywords:** *Actinobacteria*, Straw decomposition, DNA-SIP, Shotgun metagenomic sequencing, CAZymes, Soil fertility

## Abstract

**Background:**

Microbial-driven decomposition of plant residues is integral to carbon sequestration in terrestrial ecosystems. *Actinobacteria*, one of the most widely distributed bacterial phyla in soils, are known for their ability to degrade plant residues in vitro. However, their *in situ* importance and specific activity across contrasting ecological environments are not known. Here, we conducted three field experiments with buried straw in combination with microcosm experiments with ^13^C-straw in paddy soils under different soil fertility levels to reveal the ecophysiological roles of *Actinobacteria* in plant residue decomposition.

**Results:**

While accounting for only 4.6% of the total bacterial abundance, the *Actinobacteria* encoded 16% of total abundance of carbohydrate-active enzymes (CAZymes). The taxonomic and functional compositions of the *Actinobacteria* were, surprisingly, relatively stable during straw decomposition. Slopes of linear regression models between straw chemical composition and Actinobacterial traits were flatter than those for other taxonomic groups at both local and regional scales due to holding genes encoding for full set of CAZymes, nitrogenases, and antibiotic synthetases. Ecological co-occurrence network and ^13^C-based metagenomic analyses both indicated that their importance for straw degradation increased in less fertile soils, as both links between *Actinobacteria* and other community members and relative abundances of their functional genes increased with decreasing soil fertility.

**Conclusions:**

This study provided DNA-based evidence that non-dominant *Actinobacteria* plays a key ecophysiological role in plant residue decomposition as their members possess high proportions of CAZymes and as a group maintain a relatively stable presence during plant residue decomposition both in terms of taxonomic composition and functional roles. Their importance for decomposition was more pronounced in less fertile soils where their possession functional genes and interspecies interactions stood out more. Our work provides new ecophysiological angles for the understanding of the importance of *Actinobacteria* in global carbon cycling.

**Video abstract**

**Supplementary Information:**

The online version contains supplementary material available at 10.1186/s40168-021-01032-x.

## Background

Annually, more than 50,000 Tg of plant polymers are produced on earth [1]. Decomposition of these organic substances plays a pivotal role in the terrestrial ecosystem carbon balance and concomitant global change [2–4]. Plant residues mainly consist of polymers, such as cellulose, hemicelluloses, polysaccharides, and lignin [5, 6]. As soil-dwelling microorganisms are the main driving force for their decomposition [7, 8], their fate is largely determined by both the ecological (i.e., community composition and interspecies interactions) [9, 10] and physiological (i.e., encoded enzymes and their processes and pathways) [11–13] roles of soil microbial communities. Moreover, environmental conditions (e.g., soil fertility) can influence plant residue decomposition by altering community, and as a result the importance of a species can vary [14–16].

*Actinobacteria*, one of the most widely distributed phyla among soil bacteria [17], are well known for their ability to degrade plant residues [17–19]. However, the extant knowledge regarding the propensity of *Actinobacteria* to degrade plant residues is mainly based on studies with pure cultures [20, 21]. In situ conditions generally are quite different from those in the laboratory and consequently our assumptions on the *in situ* ecophysiological roles of *Actinobacteria* could be off and need validating [22]. Physiologically, *Actinobacteria* communities harbor the complete catalog of hydrolytic enzymes (e.g., *β*-glucosidase, cellobiohydrolase, ligninase, acetyl xylan esterase, arabinofuranosidase, and/or their assembled supramolecular cellulosomes) needed for plant residue decomposition [11, 17, 23]. In addition, the high C:N ratio of plant residues limits the available N for microorganisms to reproduce [24], while the nitrogen fixation ability of *Actinobacteria* may increase N availability during microbial-driven plant residue decomposition [25]. Ecologically, *Actinobacteria* can suppress competitors by synthesizing antibiotics [26]. Collectively, ecological and physiological aspects both suggest a broad adaptation of *Actinobacteria* communities to degrade plant residues and potential importance of *Actinobacteria* to residue decomposition and soil carbon sequestration. Microorganisms involved in plant residue decomposition are deterministically enriched from the surrounding soil [27]. Thus, we hypothesize that *Actinobacteria* play important ecophysiological roles in plant residue decomposition and are prevalent in environments where this process occurs.

It is well established that there is great variability of microbial taxonomic and functional composition across contrasting ecological contexts [28, 29], which highlights that the ecophysiological importance of specific microbial groups can vary across contrasting ecological environments [30]. Yet the importance of a given species in the degradation network is not a given. The current related studies are exclusively limited to small scales [11, 31, 32]. The extent of ecophysiological importance of *Actinobacteria* to degrade plant residues across contrasting ecological contexts remains elusive. It was found that the oligotrophs would dominate organic material decomposition when nutrients were limited [15, 33] and *Actinobacteria* are typically oligotrophic bacteria [34]. Thus we further hypothesize that the importance of *Actinobacteria* is enhanced in less fertile soils.

To test our hypotheses, we firstly used rice straw buried in nylon mesh bags—as a model system for plant residue decomposition—in paddy fields at three experimental sites (Chongqing (CQ), Changshu (CS), and Yingtan (YT)) with contrasting ecological contexts, especially with respect to soil fertility across subtropical China [27]. Amplicon sequencing combined with function predictions was then employed to reveal the ecological variability of the community and functional composition of several dominant straw-associated bacterial phyla (that is, *Proteobacteria*, *Firmicutes*, *Bacteroidetes*, *Actinobacteria*, and *Acidobacteria*) at both local (that is, within site) and regional (that is, across sites) scales and their co-occurrence patterns in eutrophic vs. oligotrophic soils were analyzed. Subsequently, laboratorial ^13^C-straw-based DNA stable-isotope probing (DNA-SIP) combined with shotgun metagenomic sequencing approaches were performed to characterize the physiological functional attributes of the active dominant bacterial decomposers and to verify the amplicon sequencing results at both local and regional scales under different soil fertilities.

## Results

### Dynamics of Actinobacterial taxonomic and functional profiles during straw decomposition

**Fig. 1:**
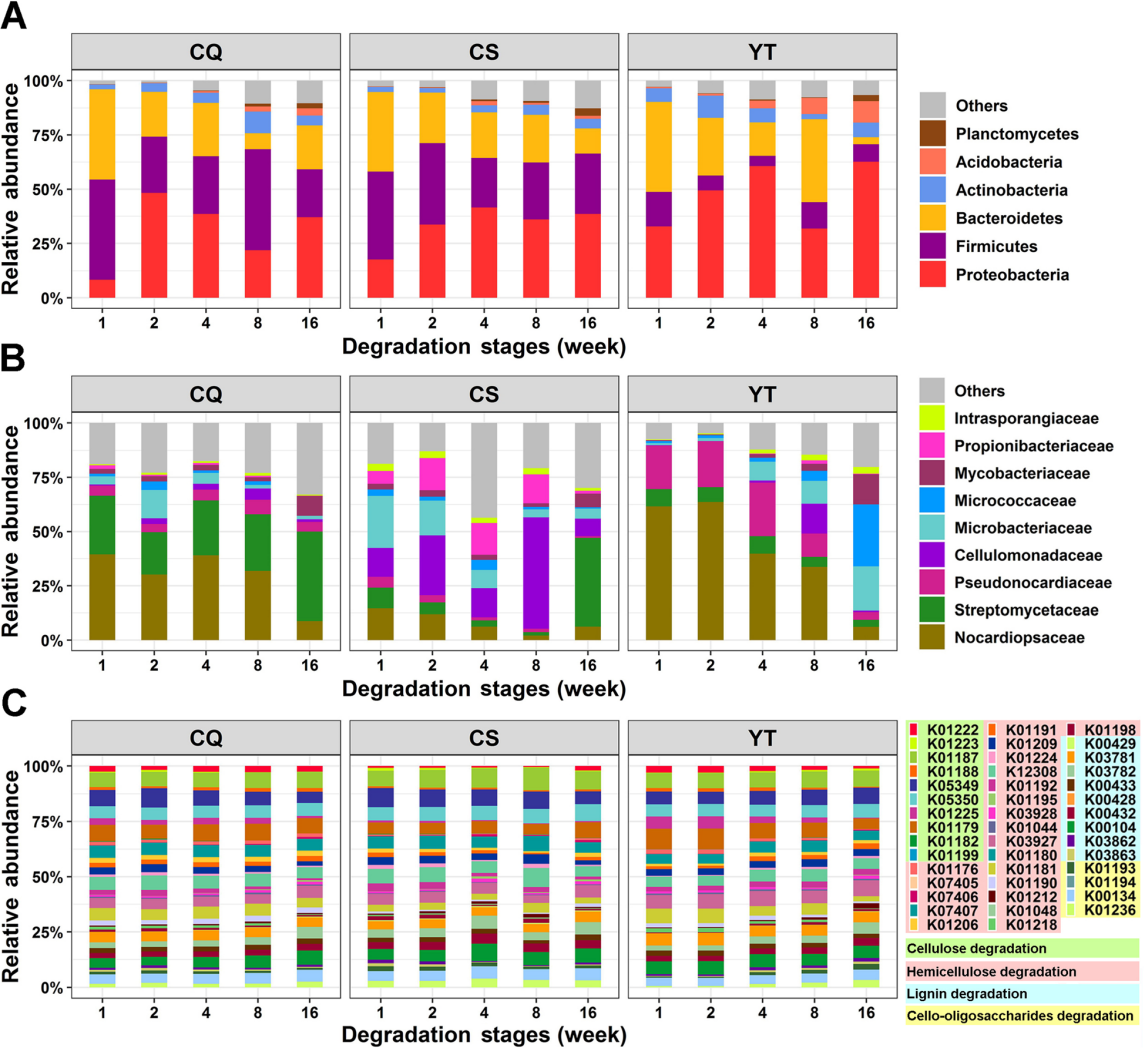
Taxonomic and functional profiles of straw decomposition bacteria at different decomposition stages. Relative abundances of dominant bacteria at the phylum level (**a**). Relative abundances of *Actinobacteria* at the family level (**b**). Relative abundances of carbohydrate-active enzymes encoding genes (one color per gene) involved in straw decomposition predicted by *Actinobacteria* (**c**). For more detailed functional gene profiles of (**c**), see Additional file 1: Table S1

The taxa in litter bags were dominated by five bacterial phyla: *Proteobacteria*, *Firmicutes*, *Bacteroidetes*, *Actinobacteria*, and *Acidobacteria* (Fig. 1a). Generally, the average relative abundance of *Proteobacteria* across decomposition stages was low at weeks 1 (19.5%) and 8 (29.9%), and higher at weeks 2 (43.8%), 4 (43.8%), and 16 (43.8%) (ANOVA, *P* < 0.05). *Firmicutes* in CQ and YT had higher relative abundance at weeks 1 (46.1% and 15.9%, respectively) and 8 (46.6% and 12.1%, respectively) and lower relative abundance at weeks 2 (25.9% and 6.8%, respectively), 4 (26.6% and 4.7%, respectively), and 16 (22.1% and 8.0%, respectively) (ANOVA, *P* < 0.05). The relative abundance of *Firmicutes* in CS was nearly stable (30.9%) across the stages. *Bacteroidetes* had the highest average relative abundance at week 1 (39.9%) (ANOVA, *P* < 0.05), while *Acidobacteria* had a higher average relative abundance in the later decomposition stages (4%) (ANOVA, *P* < 0.05). The relative abundance of *Actinobacteria* in CQ and CS was low during the first three stages (3.6% and 2.4%, respectively) and increased at later stages (7.3% and 4.5%, respectively), while the opposite was true in YT (ANOVA, *P* < 0.05). We further evaluated the changing patterns of the relative abundances of the five dominant phyla across decomposition stages at both local and regional scales (Additional file 1: Fig. S1). It was found that the relative abundance of *Actinobacteria* was less variable (with lower *F* scores, ANOVA, *P* < 0.05) than that of other phyla at both local and regional scales (except for *Firmicutes* and *Bacteroidetes* in CQ).

Specifically, the *Actinobacteria* phylum was mainly represented by 9 families in this study (Fig. 1b). *Nocardiopsaceae* and *Streptomycetaceae* were the two major families (average relative abundance: 56.5%) in CQ, while *Cellulomonadaceae*, *Microbacteriaceae*, and *Propionibacteriaceae* had higher relative abundance (average relative abundance: 50.3%) during the first four stages in CS (ANOVA, *P* < 0.05). *Nocardiopsaceae*, *Streptomycetaceae*, and *Pseudonocardiaceae* were dominant (average relative abundance: 69.0%) during the first four stages in YT, while *Microbacteriaceae*, *Micrococcaceae*, and *Mycobacteriaceae* were dominant (average relative abundance: 44.8%) at week 16. Although the taxonomic compositions of the dominant five phyla were highly variable across decomposition stages and experimental sites (with higher average *F* scores varying from 12.9 to 46.5, ANOVA, *P* < 0.05) (Fig. 1a; Additional file 1: Fig. S1), the functional composition (44 genes potentially related to plant residue decomposition, Additional file 1: Table S1) of *Actinobacteria* communities was similar (with lower average *F* score = 12.7, ANOVA, *P* < 0.05), both across stages and sites (Fig. 1c).

To evaluate the pattern of bacterial community composition change during straw decomposition at the local scale, the dominant bacteria taxa in each experimental site (that is, at the local scale) were quantified. Nonmetric multidimensional scaling analysis (NMDS) plots showed that across decomposition stages at each experimental site, the dissimilarities of taxonomic community composition of *Actinobacteria* and *Acidobacteria* were smaller than those of *Proteobacteria*, *Firmicutes*, *Bacteroidetes* (Additional file 1: Figs. S2 A-C), which was further confirmed by the results (*F* scores) of permutational multivariate analysis of variation (PERMANOVA) tests (Additional file 1: global test, *P* = 0.001, Table S2). In addition, no significant dissimilarities of the functional composition of *Actinobacteria* between stages were found in CQ (week 2 vs. week 4, *P* = 0.416; week 4 vs. week 16, *P* = 0.190), CS (week 1 vs. week 2, *P* = 0.326; week 1 vs. week 4, *P* = 0.083; week 4 vs. week 8, *P* = 0.061; week 4 vs. week 16, *P* = 0.130), and YT (week 1 vs. week 4, *P* = 0.054; week 2 vs. week 4, *P* = 0.134; week 4 vs. week 16, *P* = 0.062) (Additional file 1: Fig. S2D and Table S2).

**Fig. 2:**
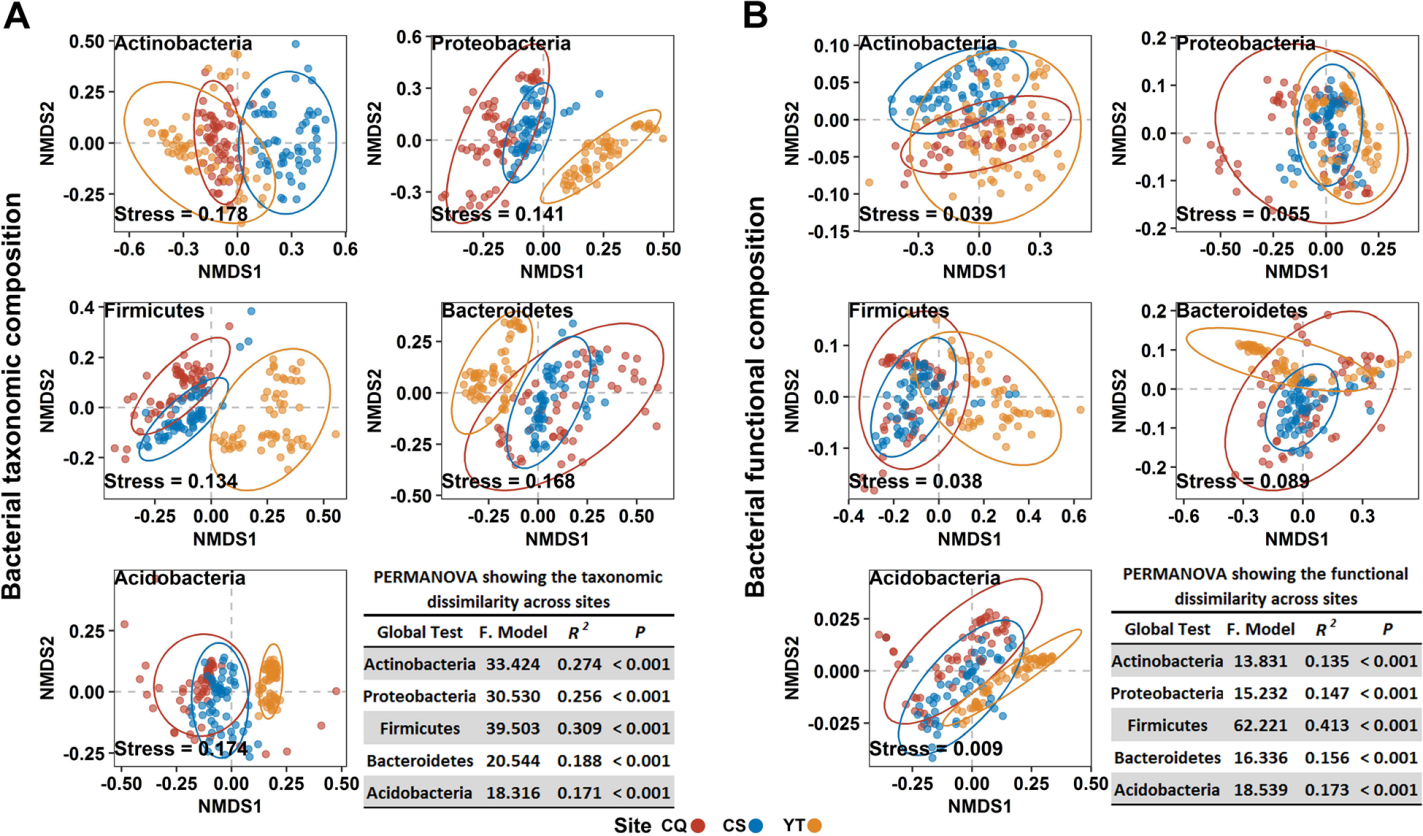
NMDS and PERMANOVA analyses of the dominant straw-associated bacterial taxonomic (**a**) and functional (**b**) composition, based on Bray-Curtis distance, across three experimental sites (*n* = 180, each plot). The circles indicate a 95% standard error of each stage

The taxonomic and functional compositions of the dominant straw decomposition bacterial taxa were further quantified to evaluate their composition at the regional scale (Fig. 2). This revealed that distinct clusters of the dominant bacteria taxa were formed in the ordination space, with significant differences being found at both taxonomic and functional levels (PERMANOVA test, *P* < 0.001, Fig. 2). Although the taxonomic composition of *Actinobacteria* was highly variable (with higher *F* score) among all experimental sites (Fig. 2a), the functional composition of *Actinobacteria* was most similar (with the lowest *F* score) at the regional scale (Fig. 2b).

### Associations among environmental factors and Actinobacterial taxonomic and functional compositions

**Fig. 3:**
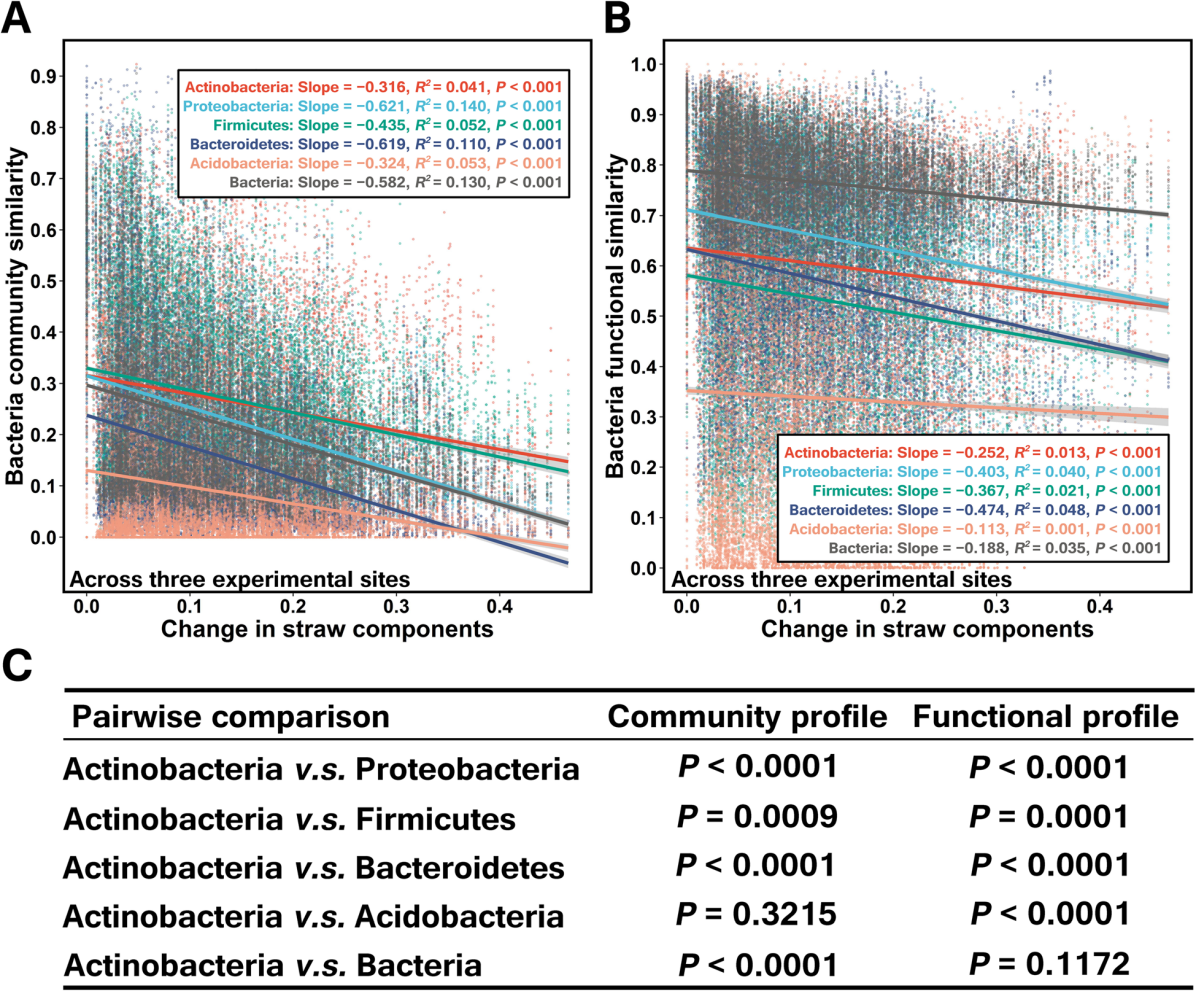
Distance matrix regressions between straw chemistry and community (**a**) and functional (**b**) composition during decomposition across three experimental sites as well as the significance of the linear regression slopes between *Actinobacteria* and other members tested by permutation tests (**c**). Horizontal axes indicate Euclidean distances based on all straw components. Linear models and associated slopes and correlation coefficients are provided on each panel

Mantel tests indicated that all soil chemical properties (e.g., available P, total K, total P, available K, SOM, and pH) were significantly correlated to soil and straw-associated bacterial community composition (Additional file 1: *P* = 0.001, Table S3). To associate the changing patterns of straw-associated bacterial community composition to straw chemistry composition at local and regional scales, straw components Euclidean distances were estimated by using all straw chemical components (that is, “all factors combined” in Additional file 1: Table S4) since the vast majority of straw components had significant correlations with the dominant bacterial taxonomic and functional composition (Additional file 1: Mantel tests, *P* < 0.05, Table S4). It was shown that straw chemistry was significantly correlated to both community and functional composition (Additional file 1: Mantel tests, *P* < 0.01, Table S4). Linear regressions further revealed significant associations between straw chemistry composition and community (Additional file 1: Fig. S3A; Fig. 3a) and functional (Additional file 1: Fig. S3B; Fig. 3b) composition at both local (Additional file 1: Fig. S3) and regional (Fig. 3) scales (*P* < 0.0001).

For the local scale, it was found that plots of *Actinobacteria* community similarity versus straw chemistry distance in CQ showed more flattened slopes (− 0.809) than other members (− 1.015~− 1.119, *P* < 0.05) (except for *Acidobacteria* (− 0.729, *P* > 0.05)). For CS, slop for *Actinobacteria* (− 1.064) was flattened than *Proteobacteria* (− 1.513, *P* < 0.0001), *Firmicutes* (− 1.155, *P* = 0.883), and *Bacteroidetes* (− 2.090, *P* < 0.0001). For YT, slop for *Actinobacteria* (− 0.879) was flattened than *Proteobacteria* (− 0.974, *P* = 0.388), *Bacteroidetes* (− 0.931, *P* = 0.903), and *Acidobacteria* (− 1.311, *P* < 0.0001) (Additional file 1: Fig. S3). Concerning plots of functional similarity versus straw chemistry distance, the slops between *Actinobacteria* and *Firmicutes* at each site were not a significant difference (*P* > 0.05). For CQ, slop for *Actinobacteria* (− 0.576) was flattened than *Proteobacteria* (− 0.751, *P* = 0.179) and *Acidobacteria* (− 1.883, *P* < 0.0001). For CS, slop for *Actinobacteria* (− 0.562) was flattened than *Proteobacteria* (− 0.789, *P* = 0.174), *Bacteroidetes* (− 1.078, *P* < 0.0001), and *Acidobacteria* (− 2.152, *P* < 0.0001). While for YT, slop for *Actinobacteria* (− 0.274) was flattened than *Proteobacteria* (− 0.442, *P* = 0.971), *Bacteroidetes* (− 0.601, *P* < 0.0001), and *Acidobacteria* (− 2.073, *P* < 0.0001) (Additional file 1: Fig. S3).

For the regional scale, plots of *Actinobacteria* community similarity versus straw chemistry distance showed more significantly (*P* < 0.001) flattened slopes (− 0.316) than other members (− 0.435~− 0.621), except for *Acidobacteria* (− 0.324, *P* = 0.322); while plots of *Actinobacteria* functional similarity versus straw chemistry distance showed more significantly (*P* < 0.001) flattened slopes (− 0.252) than *Proteobacteria* (− 0.403), *Firmicutes* (− 0.367), and *Bacteroidetes* (− 0.474) (Fig. 3).

Significantly flattened slopes of *Actinobacteria* at both community and functional level indicated that the taxonomic and functional composition of *Actinobacteria* was less variable and that they may have a relatively stable metabolic function during straw decomposition at local and regional scales.

### The importance of *Actinobacteria* in ecological co-occurrence networks of straw decomposition bacterial communities

**Fig. 4:**
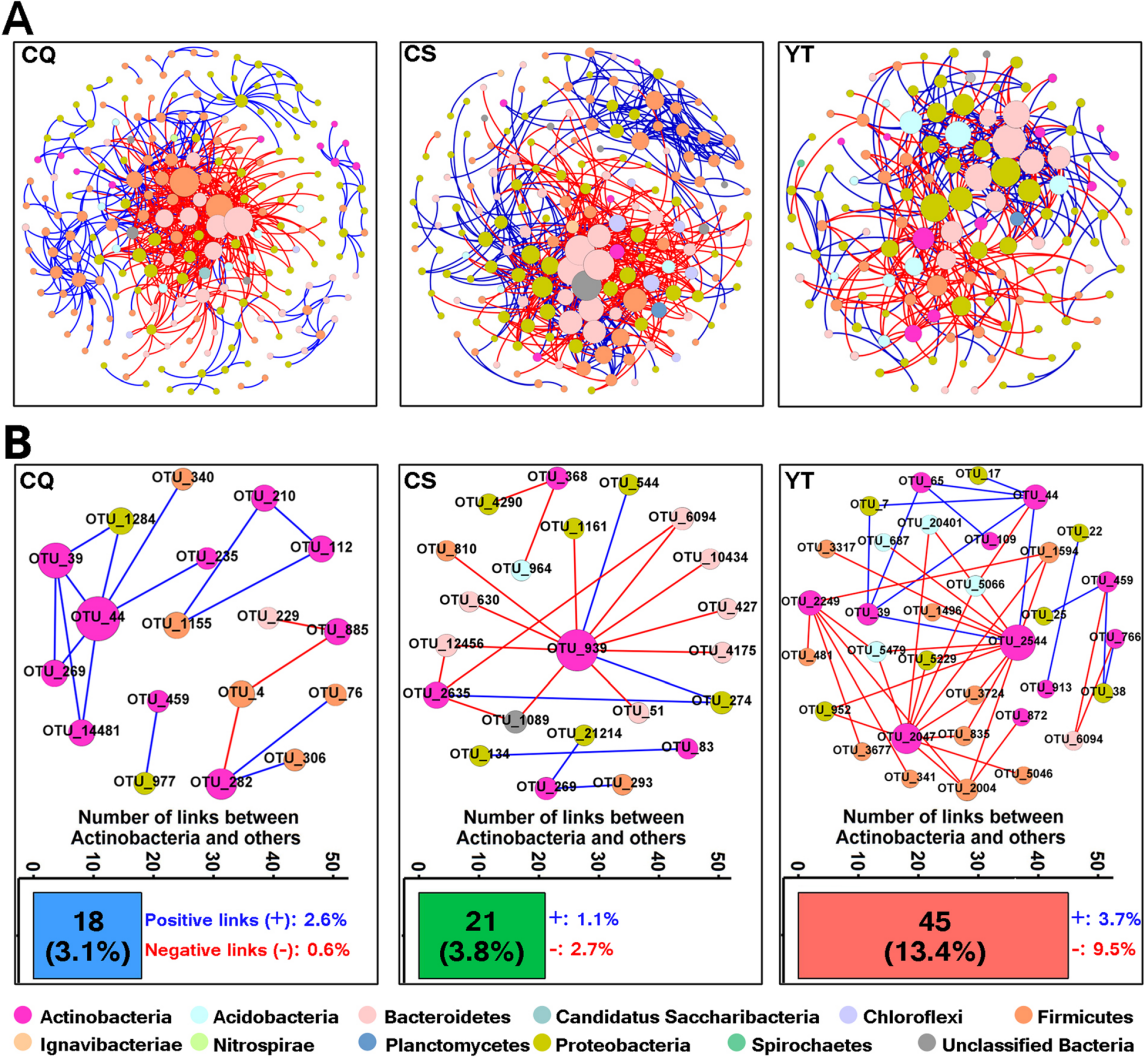
The co-occurrence network interactions of straw decomposition bacteria at each experimental site based on random matrix theory (RMT) analysis from OTU profiles (**a**). Subnetworks to visualize interactions between *Actinobacteria* and other members at each experimental site (**b**). Each node represents a bacterial phylotype (an OTU clustered at 97% identity threshold). Red and blue lines respectively represent negative and positive correlations between nodes. The size of each node is proportional to the relative abundance of OTU. For more detailed information on (**b**), see Additional file 2: Table S6

We generated phylogenetic molecular ecological networks (pMENs) for each experimental site to delineate the straw decomposition bacterial co-occurrence patterns under different soil fertilities based on correlation coefficients and *P* values for correlations (Fig. 4a). The topology indices are tabulated in Additional file 1 (Table S5). The modularity index value in each group was ranged from 0.465 to 0.578, which was higher than 0.4, indicating that they were all modularly structured co-occurrence networks [35]. The number of nodes and edges were found lower in YT than those in CQ and CS. Average degree (*avgK*; 5.321, 6.078, and 5.169 for CQ, CS, and YT respectively) measured the complexity of the network, thus YT obtained a less complex network than the other two sites (Fig. 4).

For each site, the number of links between *Actinobacteria* and other members was calculated (Fig. 4b). The number of links between *Actinobacteria* and other members in the networks was the highest (13.4% of the total links) in YT and lower in CQ and CS (3.1% and 3.8% of the total links respectively) (Fig. 4b). Similarly, the positive and negative links between *Actinobacteria* and other members were also the highest in YT (3.7% and 9.5% of the total links, respectively) and lower in CQ (2.6% and 0.6% of the total links, respectively) and CS (1.1% and 2.7% of the total links, respectively) (Fig. 4b). In network ecology, the positive links between species may suggest preferred cooperative behavior, such as metabiosis and symbiosis, while negative links between species may reflect competition (e.g., antagonism) [36]. Thus, the cooperation and competition co-occurrence patterns between *Actinobacteria* and other members may be most important in YT (Fig. 4b). More specifically, it was found that most of the species linked to *Actinobacteria* were reported to possess the functional traits involved in plant residue decomposition (Additional file 2: Table S6).

### The CAZymes repertoire of bacterial consortia revealed by DNA-SIP-based shotgun metagenomic sequencing

**Fig. 5:**
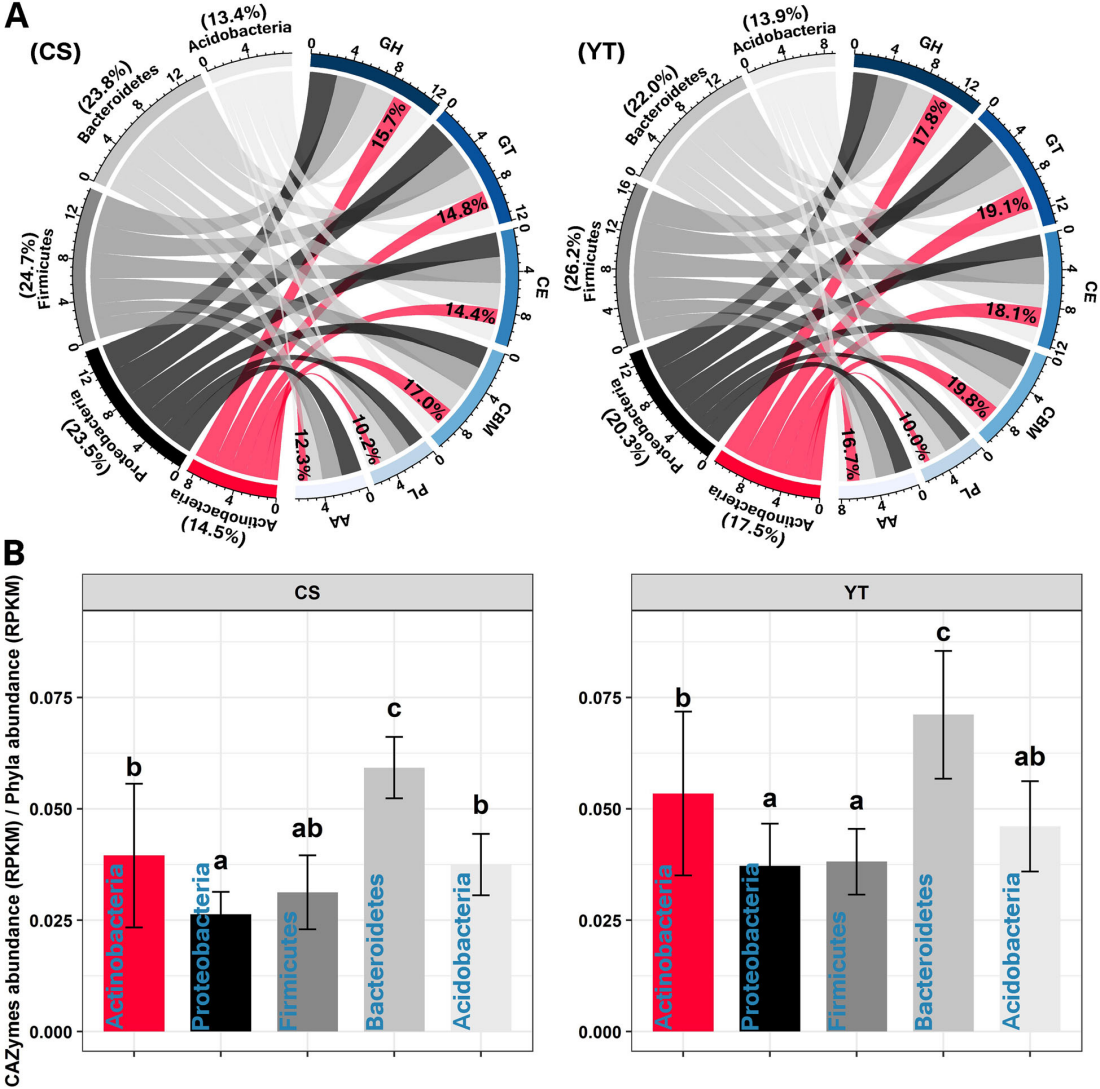
Phylogenetic distributions of CAZymes in the dominant bacterial phyla possessing CAZyme encoding-genes (**a**). The average number of CAZymes of each dominant phylum (CAZymes relative abundance/phyla relative abundance) (**b**). The relative abundances of CAZymes in (**a**) were log-transformed. *GH* glycoside hydrolase, *GT* glycosyl transferase, *PL* polysaccharide lyase, *CE* carbohydrate esterase, *CBM* carbohydrate-binding module, *AA* auxiliary activities

Because of the high functional redundancy in the microbial taxonomic pool, understanding the metabolic profiles of microbial communities is more important and necessary than just taxonomic composition when studying ecosystem functions. To better understand carbohydrate degradation in the straw decomposition ecosystem, carbohydrate-active enzymes (CAZymes) that catalyze the hydrolysis of plant residues were screened by DNA-SIP-based shotgun metagenomic sequencing. The CAZymes in the metagenome of the five dominant phyla (that is, *Proteobacteria*, *Firmicutes*, *Bacteroidetes*, *Actinobacteria*, and *Acidobacteria*) accounted for 74.5% of the total bacterial CAZymes (data not shown), which suggests that these five dominant phyla encompass the major degrading bacteria in this study. The proportions of CAZymes derived from *Actinobacteria*, *Proteobacteria*, *Firmicutes*, *Bacteroidetes*, and *Acidobacteria* were 14.5%, 23.5%, 24.7%, 23.8%, and 13.4% of the total CAZymes encoded by the five dominant phyla in CS, and were 17.5%, 20.3%, 26.2%, 22.0%, and 13.9% in YT (Fig. 5a). The CAZymes profile of the five dominant phyla were all distributed among the six CAZymes classes: glycoside hydrolase (GH), glycosyl transferase (GT), carbohydrate esterase (CE), carbohydrate binding module (CBM), polysaccharide lyase (PL), and auxiliary activities (AA). More specifically, the relative abundances of GH, GT, CE, CBM, PL, and AA derived from *Actinobacteria* were 15.7%, 14.8%, 14.4%, 17.0%, 10.2%, and 12.3%, respectively, of each CAZyme class encoded by the five dominant phyla in CS, and were 17.8%, 19.1%, 18.1%, 19.8%, 10.0%, and 16.7% in YT (Fig. 5a).

Because the taxonomic relative abundances were distributed unequally among the dominant phyla (Fig. 1a; Additional file 1: Fig. S4), the average number of CAZymes of each dominant phylum was calculated (CAZymes relative abundance/phyla relative abundance). It was found that despite *Actinobacteria* contributing lower proportions to the total CAZymes (Fig. 5a), the average number of CAZymes derived from *Actinobacteria* was significantly higher than that of *Proteobacteria* in CS, and higher than that of *Proteobacteria* and *Firmicutes* in YT (*P* < 0.05, Fig. 5b). Therefore, the relative high hydrolytic capability of *Actinobacteria* suggests that they contribute more to plant residue decomposition than those phyla. In addition to *Actinobacteria*, the high average hydrolytic capability of *Bacteroidetes* suggests that these organisms too have the potential to play important physiological roles in plant residue decomposition in soils.

### The variation distributions of the relative abundances of CAZymes in the straw decomposition consortia

**Fig. 6:**
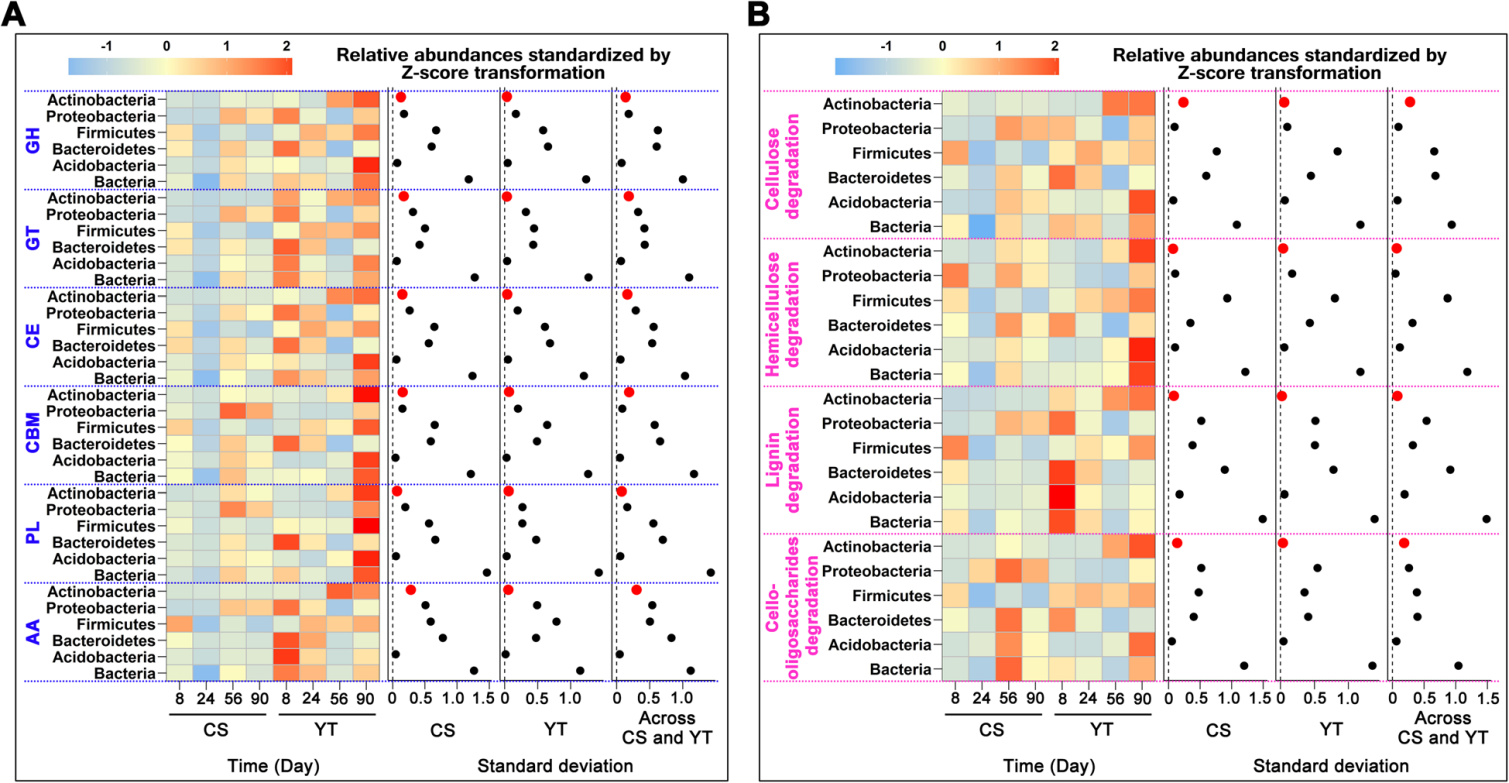
Distributions and variations (shown as standard deviation) of CAZymes (**a**) and the 44 functional genes (**b**) revealed by DNA-SIP based shotgun metagenomic sequencing that potentially related to plant residue decomposition in the dominant bacterial phyla across decomposition stages. The functional genes were merged into four groups (that is, cellulose degradation, hemicellulose degradation, lignin degradation, and cello-oligosaccharides degradation) according to substrate classification. The relative abundances of CAZymes and functional genes were Z-score transformed. *GH* glycoside hydrolase, *GT* glycosyl transferase, *PL* polysaccharide lyase, *CE* carbohydrate esterase, *CBM* carbohydrate-binding module, *AA* auxiliary activities. For more detailed functional gene profiles of (**b**), see Additional file 1: Table S1

To verify the findings of dominant bacteria functional profiles in the field experiment, the variations of the relative abundance (shown as the standard deviation of the relative abundance of CAZymes during decomposition) of CAZymes in the dominant bacteria at both local and regional scales were also evaluated (Fig. 6a). For the local scale, it was found that the average variation in CAZymes relative abundance increased in the order of *Acidobacteria* (0.034) < *Actinobacteria* (0.042) < *Proteobacteria* (0.275) < *Bacteroidetes* (0.537) < *Firmicutes* (0.556) < Bacteria (1.257) in CS (Fig. 6a), while the order was *Acidobacteria* (0.059) < *Actinobacteria* (0.175) < *Proteobacteria* (0.264) < *Firmicutes* (0.540) < *Bacteroidetes* (0.626) < Bacteria (1.414) in YT. For the regional scale, the order was *Acidobacteria* (0.054) < *Actinobacteria* (0.163) < *Proteobacteria* (0.267) < *Bacteroidetes* (0.602) < *Firmicutes* (0.605) < Bacteria (1.273). In general, the variations of the relative abundance of each class of CAZymes in the metagenome of *Actinobacteria* and *Acidobacteria* during straw decomposition at both local and regional scales were smaller than those of other members (Fig. 6a). In addition, we further evaluated the variations of the relative abundance of the 44 functional genes (Additional file 1: Table S1) potentially related to plant residue decomposition (Fig. 6b). Again, variations in the relative abundance of functional gene groups responsible for cellulose, hemicellulose, lignin, and cello-oligosaccharides degradation were lower in *Actinobacteria* and *Acidobacteria* than in other phyla, both at the local and the regional scale.

### Differences in the abundances of *Actinobacteria* and their functional genes between soils with different fertilities

**Fig. 7:**
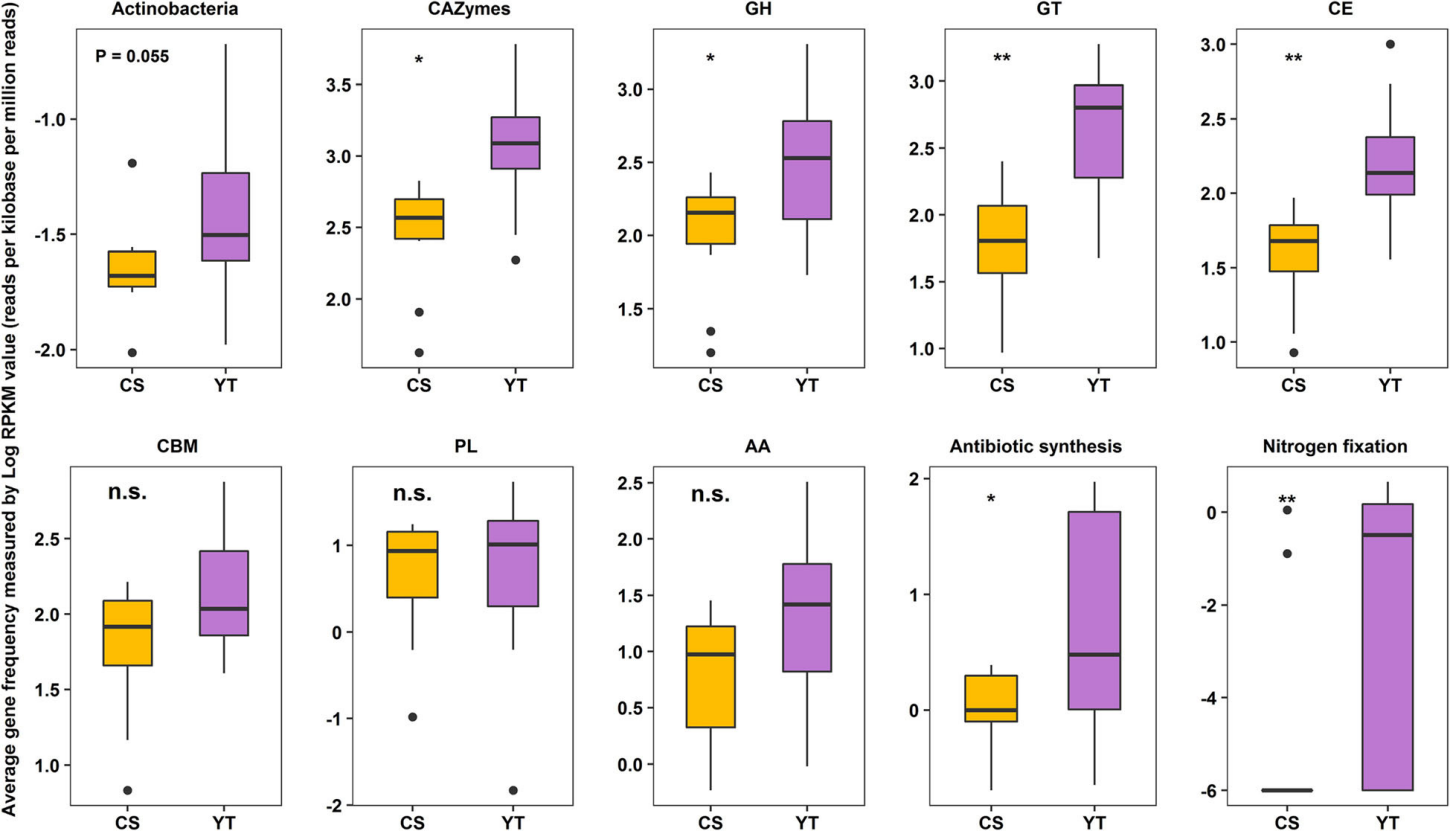
Boxplots showing the average relative abundance of *Actinobacteria* and related gene classes associated with different ecological traits in *Actinobacteria* metagenome under different soil fertilities. The relative abundances were log-transformed. *GH* glycoside hydrolase, *GT* glycosyl transferase, *PL* polysaccharide lyase, *CE* carbohydrate esterase, *CBM* carbohydrate-binding module, *AA* auxiliary activities. “*” denotes significantly different at *P* < 0.05, “**” denotes significantly different at *P* < 0.01, “n.s.” denotes *P* > 0.05

Further to the above analyses, we evaluated whether the functional genes in the metagenome can support our second hypothesis that *Actinobacteria* are more important in less fertile soils. First, we found the relative abundance of *Actinobacteria* in less fertile soil (YT) was higher than in fertile soil (CS) (*P* = 0.055, Fig. 7). Second, the relative abundances of total CAZymes and of various CAZymes classes (e.g., GH, GT, and CE) in the *Actinobacteria* metagenome were significantly higher in less fertile soil than in fertile soil (*P* < 0.05, Fig. 7). Moreover, detailed BLAST search based analysis of the genes for CAZymes in the *Actinobacteria* metagenome showed that the relative abundances of various CAZyme families with biochemical functions involved in plant residue decomposition were significantly higher in less fertile soil than in fertile soil (Additional file 3: Table S7). Third, analogous results of antibiotic synthesis genes and nitrogen fixation genes were also obtained (Fig. 7). As a potential negative control, we also evaluated the differences in the abundance of taxonomic and functional genes of another phylum—*Acidobacteria*—which has low functional variability under different soil fertilities (Fig. 6), and indeed, no significant differences were found for this phylum (Additional file 1: Fig. S5).

## Discussion

### *Actinobacteria* are not dominant but have capabilities to play an important ecophysiological role in plant residue decomposition

This study provides compelling evidence that *Actinobacteria* have the potential to play important ecophysiological roles in plant residue decomposition in soil. *Actinobacteria* contained the full set of CAZymes in higher proportion, and showed relatively low variations in taxonomic and functional composition during decomposition no matter the fertility or the climate zone where the soil was located. Low variability across spatial gradients has been attributed to the wide environmental adaptation of the functional *Actinobacteria* communities in natural ecosystems [17, 37] and to the conjecture that the genomes of the majority of the Actinobacterial taxa within a community possess all sets of related metabolic functional traits to maintain a stable presence during plant residue decomposition [38, 39]. In agreement with this theory, our study identified all classes of CAZymes and carbohydrate-active genes in the *Actinobacteria* metagenome (Figs. 5a and 6), a finding that supports and extends findings of Wang et al. [11] on Actinobacterial communities in compost heaps. Moreover, the genomes of *Actinobacteria* possess genes for nitrogen fixation and production of antibiotics (Fig. 7) which may enhance their fitness and competing to acquire carbon sources and protect against environmental perturbations [25, 26].

Even though the CAZymes derived from *Actinobacteria* were 14.5% and 17.5% of the total CAZymes for CS and YT, respectively (Fig. 5a), the average relative abundances of *Actinobacteria* only accounted for 2.5% and 6.6% of the total bacteria for CS and YT (Additional file 1: Fig. S4), which was partly not consistent with our first hypothesis that *Actinobacteria* are prevalent in plant residue decomposition. The mechanism behind this phenomenon needs further investigation. Consistently, non-dominant *Actinobacteria* metagenome encoded relatively high abundant CAZymes than other phyla (except for *Bacteroidetes* Fig. 5b). This finding is consistent with a previous study on the phylogenetic distribution of potential cellulases in bacteria, which found that the *Actinobacteria* genome harbored a high GH (a class of CAZymes) abundance for cellulose degradation [40]. Our study is highly consistent with previous work on the microbial-driven decomposition of chestnut biochar [41], as well as leaf litter [42] and wood [43], which supports the general perspective that *Actinobacteria* play important roles in plant residue decomposition, as the cell walls of nearly all green plants are made up of carbohydrates (e.g., cellulose, hemicellulose, and lignin) [44].

Although all classes of CAZymes were also found in other phyla, the high functional variability in these phyla in our study suggests that the reason behind it may differ from that of *Actinobacteria*. For example, several different subsets of taxa within these phyla may possess only subset classes of CAZymes for plant residue decomposition, which made most of the specific taxa important only on a specific time point and/or a specific site point (Fig. 6). In contrast, only a few generalists (i.e., *Actinobacteria*) that harbored all sets of enzymes for decomposition were dominant in the entire decomposition processes [11].

Also, although *Bacteroidetes* possesses the highest average CAZymes abundance (Fig. 5b), which may be taken to suggest that they thus play important physiological roles in plant residue decomposition [45], the high taxonomic and functional variability of *Bacteroidetes* (that is, their survival or life strategy may be more affected by environmental conditions) at the community level during decomposition at both local and regional scales (Figs. 3 and 6; Additional file 1: Figs. S1 and S3) implies that they are less important than *Actinobacteria* over a large spatiotemporal scale from the ecological point. Collectively, these results show that non-dominant *Actinobacteria* consistently play important ecophysiological roles throughout all straw decomposition stages by possessing high proportions of CAZymes and maintaining relative stability in taxonomic and functional composition during decomposition across spatial and environmental gradients.

### The importance of *Actinobacteria* for the decomposition of plant residues is greater in low fertility soils

Mantel tests between soil chemical properties and straw-associated bacterial community composition suggested that soil fertility (represented by available P, total K, total P, available K, and SOM) strongly affects straw-associated bacterial community composition (Additional file 1: Table S3). CAZymes revealed by shotgun metagenomic sequencing suggested that lower soil fertility (represented by YT soil) enhanced the importance of *Actinobacteria* by promoting the relative abundances of CAZymes in *Actinobacteria* (Fig. 7; Additional file 3: Table S7). This finding was consistent with previous work showing that oligotrophic microorganisms (e.g., members of the phylum *Actinobacteria* and the class *Deltaproteobacteria*) have the ability to compete for resources in a resource-limited environment [46, 47]. In addition to *Actinobacteria*, other taxa also require carbon sources to provide energy for metabolism [48]. Thus, the enhanced ability of *Actinobacteria* for plant residue decomposition in less fertile soils may provide more carbon sources for other taxa when nutrients are limited, which also suggests that *Actinobacteria* are especially important in less fertile soils.

Soil nutrient status can influence microbial interactions [15, 49]. Microorganisms can improve their ability to compete for nutrients under resource-limited conditions by streamlining into smaller cells and genomes and forming extensive species-species symbiosis among microbial members [15, 46, 50]. Co-occurrence networks provided further evidence that lower soil fertility enhanced the importance of *Actinobacteria* by increasing both the positive and negative links between *Actinobacteria* and other members (Fig. 4), which was in accordance with the streamlining theory that increases in cell-cell interactions within microbial communities are always concurrent with an increase in the competition for resources [50]. Those bacterial taxa that had the carbohydrate-active function and were positively linked to *Actinobacteria* may co-operate with *Actinobacteria* and jointly promote plant residue decomposition under lower soil fertility [51, 52]. The high relative abundance of nitrogen fixation genes in *Actinobacteria* under lower soil fertility (Fig. 7) may be one of the potential explanations for this result as these nitrogen fixation genes may increase nitrogen availability for decomposers and thus foster interspecies interactions between *Actinobacteria* and other community members for plant residue decomposition [19]. The negative links between *Actinobacteria* and bacterial taxa that had a carbohydrate-active function indicate that *Actinobacteria* may compete for carbohydrates by suppressing competitor growth [53, 54], which in turn increased the importance of *Actinobacteria* for decomposition under lower soil fertility. The higher relative abundance of antibiotic synthesis genes among *Actinobacteria* under lower soil fertility (Fig. 7) may be one of the potential explanations for their prevalence as these antibiotic synthesis genes may potentially promote antibiotic synthesis and suppress competitors for resource acquisition [26, 55]. Our results are in line with other studies indicating that under oligotrophic conditions microorganisms form associations and compete with each other to survive [15, 46]. Collectively, these results confirm our second hypothesis that *Actinobacteria* are more important in less fertile soils as they possess higher proportions of functional genes and higher proportions of interspecies interactions under lower soil fertility. However, the present data suggest but do not prove that soil fertility is the main driver behind the differences observed. Other factors such as heavy metals, trace elements, or other soil conditions may well have played a role too; further research is needed to unequivocally establish the role of soil fertility here. In addition, the final supporting evidence of all our findings in this study will require a series of studies including ones on the importance of antibiotic synthesis and the importance of nitrogen fixation by *Actinobacteria* for the organisms themselves and for the wider community. Here, we have built a case where a series of observations all point in the same direction: *Actinobacteria* potentially play an important ecophysiological role in plant residue decomposition. Further research is needed to unequivocally prove the true importance of *Actinobacteria* in plant residue degradation in this and other microbial ecosystems.

## Conclusions

DNA-based evidence presented here suggests that non-dominant *Actinobacteria* communities play important ecophysiological roles throughout all plants residue decomposition stages by possessing higher proportions of involved CAZymes and maintaining relative stability in taxonomic and functional composition during decomposition assisted by fitness-enhancing nitrogen fixing and antibiotics-producing abilities. Moreover, *Actinobacteria* are especially important in less fertile soils, as these organisms possess a relatively higher proportion of the genes involved in plant residue decomposition and are more heavily involved in interspecies interactions. Our study provides valuable insights into the important ecophysiological roles of *Actinobacteria* for carbon cycling in terrestrial ecosystems.

## Methods

### The field straw decomposition experiment

The field straw decomposition experiment was conducted at three experimental sites in CQ, CS, and YT across the subtropical zone of China (Additional file 1: Fig. S6) with a wide geographic distance (~ 1300 km) and various in soil fertilities. Details of the experimental design and site information have been described in Bao et al. [27, 56] and in Additional file 1: Tables S8 and S9 [57]. In brief, nylon litter bags (41 μm pore size, which permitted the free transfer of bacteria between paddy soils and litter bags) containing rice straw were randomly buried at 10 cm depth in a 48 m^2^ area before rice cultivation. Then, the bags and their adjacent soil samples with 12 replicates were collected at 1, 2, 4, 8, and 16 weeks after they were buried. In total, 180 straw samples and 180 soil samples were used in this investigation. Subsamples of straw for decomposition and chemistry assays were stored at − 20 °C, and subsamples for DNA extraction were stored at − 80 °C. We note that these samples were also used in previous studies [27, 56]. The straw decomposition ratio was determined according to the methods described in Bao et al. [27]. The straw decomposition ratios of CQ, CS, and YT were supplied in Additional file 1: Table S10 [27].

### Straw chemical properties measurement

The straw components of cellulose, hemicellulose, and lignin were determined according to validated methods described by Van Soest [58] with some modifications [59]. In brief, thermogravimetric analysis with 1 g straw sample was performed using the crude fiber extractor FIWE 3 (Velp Scientifica, Italy). The straw sample was boiled in neutral detergent solution (sodium dodecyl sulfate, EDTA, pH 7.0, 100 mL) for 1 h, then washed with hot water and acetone, and finally dried at 105 °C for 12 h. Neutral detergent fiber fraction was weighed. The same procedure except samples boiled in acidic detergent solution (cetyltrimethyl ammonium bromide in 1 N H_2_SO_4_, 100 mL) was taken to measure acidic detergent fiber fraction. The cellulose was extracted 4 h by adding 20 mL of 72% sulfuric acid to the residue. Then, the sample was thoroughly rinsed with hot water and finally with acetone then dried at 105 °C for 12 h. Acid detergent lignin fraction was weighed. Then the cellulose, hemicellulose, and lignin content were calculated. The water-soluble polysaccharides (WSP) was extracted at 70 °C for 30 min with distilled water (straw:water = 1:10). Insoluble material was removed by filtration. After the extracted solution was roto-evaporated to 25 mL, crude polysaccharide was precipitated by ethanol. Finally, this was dried and weighed for calculating the content of WSP [60].

### Amplicon high-throughput sequencing and data processing

To test our hypotheses, we determined the straw decomposition bacterial communities by Illumina sequencing of 16S rRNA genes. Genomic DNA was extracted using a FastDNA® SPIN Kit for soil (MP Biomedicals, Santa Ana, CA) with a negative control following the manufacturer’s instructions. The 16S rRNA gene V4-V5 fragments were amplified using primer pairs 519F (5′-CAGCMGCCGCGGTAATWC-3′) and 907R (5′-CCGTCAATTCMTTTRAGTTT-3′). The 5-bp bar-coded oligonucleotides were fused to the forward primer to distinguish different samples. The procedures of PCR reaction, amplicon high-throughput sequencing libraries preparing, and data processing were fully described in Bao et al. [27]. In total, 6,528,688 quality bacterial 16S rRNA gene sequences were obtained, and between 8081 and 39,732 sequences per sample (with a median value of 17,228 sequences per sample). Then, all samples were randomly rarified to 8000 sequences for downstream analyses, which was extremely close to the minimum sequence number for all samples.

### Microbial community analyses

Functional characteristics of bacterial communities during straw decomposition in the field experiment were analyzed by phylogenetic investigation of communities by reconstruction of unobserved states (PICRUSt) [61]. In brief, a PICRUSt-compatible operational taxonomic unit (OTU) table was constructed using the closed-reference OTU-picking protocol in Quantitative Insights Into Microbial Ecology (QIIME, USA) [62] against the Greengenes database, then the ancestral states in the reference tree were reconstructed and the gene function spectrum of tips that lack sequenced genomes were predicted by identifying the nearest corresponding ancestor, and function prediction was compared to the annotated whole-genome sequencing metagenome across KEGG Orthology. Finally, the gene functions were identified. The straw-associated bacterial community composition was visualized by NMDS based on Bray-Curtis distance. PERMANOVA [63] was conducted to test for statistically significant differences in community composition among stages, using R software (the “vegan” package [64], Version 2.2-1).

### Molecular ecological network analysis

To reveal the co-occurrence patterns in straw-associated bacterial communities in the field experiment, phylogenetic molecular ecological networks (pMENs) in CQ, CS, and YT were constructed using the random matrix theory (RMT)-based network approach [65]. The pMEN construction and analysis were performed with the on-line pipeline of Deng et al. [66]. Network parameters, such as total nodes, total links, average degree, geodesic efficiency, harmonic geodesic distance, and transitivity were used to evaluate the topological structure of the co-occurrence networks. The constructed networks for bacterial communities involved in straw decomposition were visualized using Gephi software [67].

### ^13^C-straw amended microcosms and DNA stable isotope probing gradients

To further verify our hypotheses, we determined the straw decomposition bacterial community and functional composition by DNA stable-isotope probing (DNA-SIP) microcosms and metagenomic shotgun sequencing. Two soils with different fertility (as representatives of different soil fertility levels) were sampled from the abovementioned rice field experimental sites in CS and YT. Ten grams of soil were added per serum bottle (120 mL, 10 cm in height) and then pre-incubated for 3 days in the dark at 27 °C. Soil moisture was then adjusted to 60% of the maximum water holding capacity. After that, 0.1 g of ^13^C-labeled rice straw (ca. 70 at%) was added to each bottle (^13^C) (the ^13^C-labeled rice straw was obtained from the previous study [68]). The serum bottles were flushed with N_2_ for 10 min to obtain an anaerobic condition, and then bottles were sealed and incubated in the dark at 27 °C for sampling. Due to ^12^C controls were extremely useful to identify the “heavy” DNA fractions from SIP incubations [69], thus bottles amended with natural ^12^C-rice straw (ca. 1.08% of ^13^C to ΣC) (^12^C) were established as the control of the ^13^C treatment. Each treatment was replicated three times. Soil samples were respectively collected at 8, 24, 56, and 90 days after they were incubated, and a total of 12 soil samples were collected for each experimental site. In total, there were 24 soil samples in this investigation.

DNA stable isotope fractionation was performed as described by Jia and Conrad [70]. In brief, the gradient fractionation of total DNA (3.0 μg) extract from the soil of all SIP microcosms was conducted with an initial CsCl buoyant density of 1.720 g/mL; then the solutions were centrifugated at 177,000 g for 44 h under vacuum using a Beckman optima TLX (Beckman Coulter, Inc., Palo Alto, CA, USA). After ultracentrifugation, the solution was immediately separated from bottom to top into 15 equal fractions using a calibrated infusion pump (New Era Pump System, Inc., Farmingdale, NY, USA). DNA was separated from CsCl by PEG 6000 precipitation and dissolved in TE buffer.

The copy numbers of the bacterial 16S rRNA gene V4-V5 fragments in each DNA fractions were quantified by real-time quantitative PCR (qPCR) according to the previously described procedures [19]. Shifts in the bacterial 16S rRNA gene copy numbers of the isotopically fractionated DNA gradients were shown in Additional file 1: Fig. S7. DNA fractions with green circles in the figure were defined as “heavy” DNA fractions.

### Shotgun metagenomic sequencing of DNA in “heavy” buoyant fractions

To generate sufficient DNA for preparing of shotgun metagenomic sequencing library, “heavy” DNA fractions of ^13^C-straw treatments were amplified by the multiple displacement amplification (MDA) method using REPLI-g Single Cell (sc) Kit (#150345; QIAGEN, Hilden, Germany) according to standard protocols of the manufacturer. Negative controls were conducted following the same protocol. Then, DNA was fragmented to ~ 300 bp using Covaris M220 (Gene Company Limited, China) for paired-end library construction. TruSeq™ DNA Sample Prep Kit (Illumina, San Diego, CA, USA) was used to prepare the paired-end library. Adapters were ligated to the Blunt-end fragments. The HiSeq 3000/4000 SBS Kits, HiSeq 3000/4000 PE Cluster Kit, and Illumina HiSeq 4000 platform (Illumina Inc., San Diego, CA, USA) were used for sequencing at Majorbio Bio-Pharm Technology Co., Ltd. (Shanghai, China) according to standard protocols of the manufacturer. Totally, ~ 10 Gbp paired-end Illumina data were obtained for each sample. All the raw metagenomics datasets have been deposited into the NCBI SRA database (accession no., PRJNA669350).

Adaptors were stripped using SeqPrep (https://github.com/jstjohn/SeqPrep). Low-quality reads (length < 50 bp or with a quality value < 20 or having N bases) were filtered with Sickle (https://github.com/najoshi/sickle) using default parameters. The de Bruijn graph-based assembler SOAPdenovo (http://soap.genomics.org.cn, Version 1.06) was employed to assemble short reads (K-mers range 47-97, step-10). K-mers varying from 1/3 to 2/3 of read lengths were then tested for all samples. Scaffolds with a length > 500 bp were retained for statistical tests; the quality and quantity of scaffolds generated were evaluated by each assembly and chose the best K-mer, which yielded the maximum value of N50 and N90 and the minimum scaffold number, respectively. Scaffolds with a length > 500 bp were then extracted and broken into contigs without gaps. These contigs were used for further gene prediction and annotation.

Open reading frames (ORFs) from each metagenomic sample were predicted using MetaGene (http://metagene.cb.k.u-tokyo.ac.jp/). The predicted ORFs with length being or over 100 bp were retrieved and translated to amino acid sequences using the NCBI translation table (http://www.ncbi.nlm.nih.gov/Taxonomy/taxonomyhome.html/index.cgi?chapter=tgencodes#SG1). All sequences from gene sets with a 95% sequence identity (90% coverage) were clustered as the non-redundant gene catalog by the CD-HIT (http://www.bioinformatics.org/cd-hit/). Reads after quality control were mapped to the representative genes with 95% identity using SOAPaligner (http://soap.genomics.org.cn/), and gene abundance in each sample was evaluated. BLASTP (Version 2.2.28+, http://blast.ncbi.nlm.nih.gov/Blast.cgi) was employed for taxonomic annotations by aligning non-redundant gene catalogs against the NCBI NR database with an *e* value cutoff of 1e^−5^. The cluster of orthologous groups of proteins (COG) for the ORFs annotation was performed using BLASTP against the eggNOG database (v4.5) with an *e* value cutoff of 1e^−5^. The KEGG pathway annotation was conducted using the BLASTP search (Version 2.2.28+) against the Kyoto Encyclopedia of Genes and Genomes database (http://www.genome.jp/keeg/) with an *e* value cutoff of 1e^−5^. For the analysis of carbohydrate-active enzymes (CAZymes) in the dominant bacterial phyla, the non-redundant gene catalogs were firstly taxonomically assigned by BLASTP as mentioned above, then the CAZymes functions of the non-redundant gene catalogs with taxonomic assignment were annotated using hmmscan (http://hmmer.janelia.org/search/hmmscan) against CAZy database V5.0 (http://www.cazy.org/) with an *e* value cutoff of 1e^−5^. All the shotgun metagenomic sequencing data were normalized with the reads assigned per kilobase of target per million mapped reads (RPKM) method [71].

### Statistical analysis

Mantel tests were conducted between environmental factors (soil chemical properties and/or straw chemical components) and bacterial community (taxonomic and/or functional composition) using R software (the “vegan” package [64], version 2.2-1). Linear regressions between Bray-Curtis distance and changes in straw chemical components were conducted to determine the relationship between bacterial communities and straw chemical components. The changes in straw chemical components were calculated using 4 chemical components of straw samples (Additional file 1: Table S11) [56] based on Euclidean distances. The significance of the linear regression slopes within phylum and between *Actinobacteria* and other members were tested by permutation tests. Separation of mean values among different samples was evaluated with one-way ANOVA followed by post-hoc Tukey’s HSD tests using the IBM Statistical Product and Service Solutions (SPSS) Statistics for Windows (Version 13). The difference of *P* < 0.05 was considered significant.

## Supplementary Information

**Additional file 1. **“Important ecophysiological roles of non-dominant *Actinobacteria* in plant residue decomposition, especially in less fertile soils”. **Table S1.** Carbohydrate-active enzymes encoding genes involved in plant residue decomposition. **Table S2.** PERMANOVA showing the dissimilarities of the dominant straw-associated bacterial taxonomic and *Actinobacteria* functional composition between different decomposition stages, based on Bray-Curtis distance, in CQ, CS, and YT. **Table S3.** Mantel test between soil chemical properties and soil and straw-associated bacterial community composition across three experimental sites. **Table S4.** Mantel test between straw chemical components and the dominant bacterial taxonomic and functional composition at local and regional scales. **Table S5.** Topological properties of networks of straw decomposition bacterial communities at each experimental site. **Table S8.** Description of three field experimental sites. **Table S9.** Soil chemical properties of three experimental sites. **Table S10.** Straw decomposition ratios over 16-week decomposition stages. **Table S11.** Concentration of straw chemical components over 16-week decomposition stages. **Figure S1.** Relative abundances of dominant bacteria across decomposition stages at the phylum level in CQ (A), CS (B), YT (C), and across three experimental sites (D). **Figure S2.** Nonmetric multidimensional (NMDS) analysis of the dominant straw-associated bacterial taxonomic (A-C) and *Actinobacteria* functional (D) composition between different decomposition stages, based on Bray-Curtis distance, in CQ, CS, and YT (n = 60, each plot). The circles indicate a 95% standard error of each stage. **Figure S3.** Distance matrix regressions between straw chemistry and community (A) and functional (B) composition during decomposition within each experimental site. The slops and correlation coefficients of linear models are provided (C) as well as the significance of the linear regression slopes between *Actinobacteria* and other members tested by permutation tests (D). Horizontal axes indicate Euclidean distances based on all straw components. “*” denotes that the slopes were significantly less than zero by permutation tests at *P* < 0.0001. **Figure S4.** Relative abundances of dominant bacteria at the phylum level revealed by DNA-SIP based shotgun metagenomic sequencing. **Figure S5.** Boxplots showing the average relative abundance of *Acidobacteria* and related gene classes revealed by DNA-SIP based shotgun metagenomic sequencing that associated with different ecological traits in *Acidobacteria* metagenome under different soil fertility. The relative abundances were log-transformed. GH: Glycoside hydrolase, GT: glycosyl transferase, PL: polysaccharide lyase, CE: carbohydrate esterase, CBM: carbohydrate-binding module, and AA: auxiliary activities. “n.s.” denotes *P* > 0.05. **Figure S6.** Locations of the three experimental sites (CQ, CS, and YT). **Figure S7.** Distributions of the copy numbers of bacterial 16S rRNA gene across the buoyant densities of the DNA gradients isolated from soil samples treated with ^13^C- or with ^12^C-straw in CS (A) and YT (B). DNA fractions with green circles were defined as “heavy” genomic DNA fractions and were then subjected to shotgun metagenomic sequencing.

**Additional file 2. **“Important ecophysiological roles of non-dominant *Actinobacteria* in plant residue decomposition, especially in less fertile soils”. **Table S6.** Taxonomic information of species linked to *Actinobacteria* revealed by co-occurrence network analyses.

**Additional file 3. **“Important ecophysiological roles of non-dominant *Actinobacteria* in plant residue decomposition, especially in less fertile soils”. **Table S7.** Detailed analysis of CAZyme functions in *Actinobacteria* metagenome revealed by DNA-SIP based shotgun metagenomic sequencing.

## Data Availability

The sequences of 16S rRNA genes amplicons of field experiments are deposited into the NCBI SRA database (accession no., PRJNA591776). The shotgun metagenome datasets of microcosm experiments are available in the NCBI SRA database (accession no., PRJNA669350).

## Abbreviations

CAZymes: Carbohydrate-active enzymes
COG: Cluster of orthologous groups of proteins
CQ: Chongqing
CS: Changshu
DNA-SIP: DNA stable-isotope probing
MDA: Multiple displacement amplification
NMDS: Nonmetric multidimensional scaling analysis
ORF: Open reading frame
OTU: Operational taxonomic unit
PERMANOVA: Permutational multivariate analysis of variation
PICRUSt: Phylogenetic investigation of communities by reconstruction of unobserved states
pMEN: Phylogenetic molecular ecological network
QIIME: Quantitative Insights Into Microbial Ecology
qPCR: Quantified by real-time quantitative PCR
RMT: Random matrix theory
RPKM: Reads assigned per kilobase of target per million mapped reads
SPSS: Statistical product and service solutions
WSP: Water-soluble polysaccharides
YT: Yingtan
