# Supplementary material for: Important ecophysiological roles of non-dominant Actinobacteria in plant residue decomposition, especially in less fertile soils
Source: Microbiome. 2021 Apr 7;9:84. doi: 10.1186/s40168-021-01032-x (PMC8028251; doi:10.1186/s40168-021-01032-x)
Supplement: Supplementary file 2 — Additional file 1. “Important ecophysiological roles of non-dominant Actinobacteria in plant residue decomposition, especially in less fertile soils”. Table S1. Carbohydrate-active enzymes encoding genes involved in plant residue decomposition. Table S2. PERMANOVA showing the dissimilarities of the dominant straw-associated bacterial taxonomic and Actinobacteria functional composition between different decomposition stages, based on Bray-Curtis distance, in CQ, CS, and YT. Table S3. Mantel test between soil chemical properties and soil and straw-associated bacterial community composition across three experimental sites. Table S4. Mantel test between straw chemical components and the dominant bacterial taxonomic and functional composition at local and regional scales. Table S5. Topological properties of networks of straw decomposition bacterial communities at each experimental site. Table S8. Description of three field experimental sites. Table S9. Soil chemical properties of three experimental sites. Table S10. Straw decomposition ratios over 16-week decomposition stages. Table S11. Concentration of straw chemical components over 16-week decomposition stages. Figure S1. Relative abundances of dominant bacteria across decomposition stages at the phylum level in CQ (A), CS (B), YT (C), and across three experimental sites (D). Figure S2. Nonmetric multidimensional (NMDS) analysis of the dominant straw-associated bacterial taxonomic (A-C) and Actinobacteria functional (D) composition between different decomposition stages, based on Bray-Curtis distance, in CQ, CS, and YT (n = 60, each plot). The circles indicate a 95% standard error of each stage. Figure S3. Distance matrix regressions between straw chemistry and community (A) and functional (B) composition during decomposition within each experimental site. The slops and correlation coefficients of linear models are provided (C) as well as the significance of the linear regression slopes between Actinobacteria and othe [file 40168_2021_1032_MOESM2_ESM.docx]

**Table S1.** Carbohydrate-active enzymes encoding genes involved in plant residue decomposition ([Zheng et al., 2018](#_ENREF_4)).

| **Straw components** | **KEGG orthology** | **KEGG description** | **EC** |
| --- | --- | --- | --- |
| Cellulose | K01222 | 6-phospho-beta-glucosidase | [EC:3.2.1.86] |
|  | K01223 | 6-phospho-beta-glucosidase | [EC:3.2.1.86] |
|  | K01187 | alpha-glucosidase | [EC:3.2.1.20] |
|  | K01188 | beta-glucosidase | [EC:3.2.1.21] |
|  | K05349 | beta-glucosidase | [EC:3.2.1.21] |
|  | K05350 | beta-glucosidase | [EC:3.2.1.21] |
|  | K01225 | cellulose 1,4-beta-cellobiosidase | [EC:3.2.1.91] |
|  | K01179 | endoglucanase | [EC:3.2.1.4] |
|  | K01182 | oligo-1,6-glucosidase | [EC:3.2.1.10] |
|  | K01199 | glucan endo-1,3-beta-D-glucosidase | [EC:3.2.1.39] |
| Hemicellulose | K01176 | alpha-amylase | [EC:3.2.1.1] |
|  | K07405 | alpha-amylase | [EC:3.2.1.1] |
|  | K07406 | alpha-galactosidase | [EC:3.2.1.22] |
|  | K07407 | alpha-galactosidase | [EC:3.2.1.22] |
|  | K01206 | alpha-L-fucosidase | [EC:3.2.1.51] |
|  | K01209 | alpha-N-arabinofuranosidase | [EC:3.2.1.55] |
|  | K01224 | arabinogalactan endo-1,4-beta-galactosidase | [EC:3.2.1.89] |
|  | K01190 | beta-galactosidase | [EC:3.2.1.23] |
|  | K12308 | beta-galactosidase | [EC:3.2.1.23] |
|  | K01192 | beta-mannosidase | [EC:3.2.1.25] |
|  | K01195 | beta-glucuronidase | [EC:3.2.1.31] |
|  | K03928 | carboxylesterase | [EC:3.1.1.1] |
|  | K01044 | carboxylesterase | [EC:3.1.1.1] |
|  | K03927 | carboxylesterase type B | [EC:3.1.1.1] |
|  | K01180 | endo-1,3(4)-beta-glucanase | [EC:3.2.1.6] |
|  | K01181 | endo-1,4-beta-xylanase | [EC:3.2.1.8] |
|  | K01190 | beta-galactosidase | [EC:3.2.1.23] |
|  | K01212 | levanase | [EC:3.2.1.65] |
|  | K01048 | lysophospholipase | [EC:3.1.1.5] |
|  | K01218 | mannan endo-1,4-beta-mannosidase | [EC:3.2.1.78] |
|  | K01198 | xylan 1,4-beta-xylosidase | [EC:3.2.1.37] |
| Lignin | K00104 | glycolate oxidase | [EC:1.1.3.15] |
|  | K00429 | catalase | [EC:1.11.1.6] |
|  | K03781 | catalase | [EC:1.11.1.6] |
|  | K03782 | catalase/peroxidase | [EC:1.11.1.6; 1.11.1.7] |
|  | K00433 | chloride peroxidase | [EC:1.11.1.10] |
|  | K00428 | cytochrome c peroxidase | [EC:1.11.1.5] |
|  | K00432 | glutathione peroxidase | [EC:1.11.1.9] |
|  | K03862 | vanillate monooxygenase | [EC:1.14.13.82] |
|  | K03863 | vanillate monooxygenase | [EC:1.14.13.82] |
| Cello-oligosaccharides | K01193 | beta-fructofuranosidase | [EC:3.2.1.26] |
|  | K01194 | alpha,alpha-trehalase | [EC:3.2.1.28] |
|  | K00134 | glyceraldehyde 3-phosphate dehydrogenase | [EC:1.2.1.12] |
|  | K01236 | maltooligosyltrehalose trehalohydrolase | [EC:3.2.1.141] |

**Table S2.** PERMANOVA showing the dissimilarities of the dominant straw-associated bacterial taxonomic and *Actinobacteria* functional composition between different decomposition stages, based on Bray-Curtis distance, in CQ, CS, and YT.

| **Site** | **Pairs** | **Bray-Curtis dissimilarity based on *Actinobacteria* community** | | | **Bray-Curtis dissimilarity based on *Proteobacteria* community** | | | **Bray-Curtis dissimilarity based on *Firmicutes* community** | | | **Bray-Curtis dissimilarity based on *Bacteroidetes* community** | | | **Bray-Curtis dissimilarity based on *Acidobacteria* community** | | | **Bray-Curtis dissimilarity based on *Actinobacteria* functional composition** | | |
| --- | --- | --- | --- | --- | --- | --- | --- | --- | --- | --- | --- | --- | --- | --- | --- | --- | --- | --- | --- |
|  |  | **F. Model** | **R^2^** | **Pr (>F)** | **F. Model** | **R^2^** | **Pr (>F)** | **F. Model** | **R^2^** | **Pr (>F)** | **F. Model** | **R^2^** | **Pr (>F)** | **F. Model** | **R^2^** | **Pr (>F)** | **F. Model** | **R^2^** | **Pr (>F)** |
| CQ | Global test | 12.9 | 0.5 | 0.001 | 16.2 | 0.5 | 0.001 | 14.9 | 0.5 | 0.001 | 15.5 | 0.5 | 0.001 | 4.3 | 0.2 | 0.001 | 20.6 | 0.6 | 0.001 |
|  | CQ_1 W vs CQ_16 W | 13.7 | 0.4 | 0.001 | 22.6 | 0.5 | 0.00 | 25.5 | 0.5 | 0.001 | 21.7 | 0.5 | 0.001 | 6.5 | 0.2 | 0.001 | 21.4 | 0.5 | 0.001 |
|  | CQ_1 W vs CQ_2 W | 6.7 | 0.2 | 0.002 | 16.8 | 0.4 | 0.001 | 7.5 | 0.3 | 0.001 | 24.2 | 0.5 | 0.001 | 0.9 | 0.0 | 0.753 | 18.0 | 0.5 | 0.001 |
|  | CQ_1 W vs CQ_4 W | 5.8 | 0.2 | 0.005 | 14.8 | 0.4 | 0.001 | 16.5 | 0.4 | 0.001 | 16.5 | 0.4 | 0.001 | 2.4 | 0.1 | 0.001 | 14.1 | 0.4 | 0.003 |
|  | CQ_1 W vs CQ_8 W | 32.2 | 0.6 | 0.001 | 18.2 | 0.5 | 0.001 | 34.0 | 0.6 | 0.001 | 33.0 | 0.6 | 0.001 | 6.9 | 0.2 | 0.001 | 61.8 | 0.7 | 0.001 |
|  | CQ_16 W vs CQ_2 W | 14.0 | 0.4 | 0.001 | 23.7 | 0.5 | 0.001 | 13.6 | 0.4 | 0.001 | 13.7 | 0.4 | 0.001 | 6.0 | 0.2 | 0.001 | 4.8 | 0.2 | 0.018 |
|  | CQ_16 W vs CQ_4 W | 9.2 | 0.3 | 0.001 | 17.6 | 0.4 | 0.001 | 6.9 | 0.2 | 0.001 | 11.0 | 0.3 | 0.001 | 6.0 | 0.2 | 0.001 | 1.8 | 0.1 | 0.190 |
|  | CQ_16 W vs CQ_8 W | 18.5 | 0.5 | 0.001 | 15.8 | 0.4 | 0.001 | 12.4 | 0.4 | 0.001 | 13.0 | 0.4 | 0.001 | 6.8 | 0.2 | 0.001 | 45.6 | 0.7 | 0.001 |
|  | CQ_2 W vs CQ_4 W | 3.5 | 0.1 | 0.011 | 5.0 | 0.2 | 0.001 | 8.3 | 0.3 | 0.001 | 6.0 | 0.2 | 0.001 | 1.8 | 0.1 | 0.001 | 0.7 | 0.0 | 0.416 |
|  | CQ_2 W vs CQ_8 W | 24.6 | 0.5 | 0.001 | 17.3 | 0.4 | 0.001 | 21.5 | 0.5 | 0.001 | 18.3 | 0.5 | 0.001 | 6.1 | 0.2 | 0.001 | 42.1 | 0.7 | 0.001 |
|  | CQ_4 W vs CQ_8 W | 10.3 | 0.3 | 0.001 | 9.2 | 0.3 | 0.001 | 10.4 | 0.3 | 0.001 | 9.8 | 0.3 | 0.001 | 4.1 | 0.2 | 0.001 | 20.6 | 0.5 | 0.001 |
| CS | Global test | 10.6 | 0.4 | 0.001 | 15.8 | 0.5 | 0.001 | 14.7 | 0.5 | 0.001 | 20.0 | 0.6 | 0.001 | 3.6 | 0.2 | 0.001 | 5.5 | 0.3 | 0.001 |
|  | CS_1 W vs CS_16 W | 13.0 | 0.4 | 0.001 | 27.5 | 0.6 | 0.001 | 29.2 | 0.6 | 0.001 | 24.8 | 0.5 | 0.001 | 3.9 | 0.2 | 0.001 | 5.7 | 0.2 | 0.013 |
|  | CS_1 W vs CS_2 W | 3.3 | 0.1 | 0.001 | 12.6 | 0.4 | 0.001 | 14.6 | 0.4 | 0.001 | 19.8 | 0.5 | 0.001 | 1.6 | 0.1 | 0.013 | 1.0 | 0.0 | 0.326 |
|  | CS_1 W vs CS_4 W | 6.3 | 0.2 | 0.001 | 14.5 | 0.4 | 0.001 | 15.4 | 0.4 | 0.001 | 21.2 | 0.5 | 0.001 | 2.4 | 0.1 | 0.001 | 2.5 | 0.1 | 0.083 |
|  | CS_1 W vs CS_8 W | 16.5 | 0.4 | 0.001 | 28.2 | 0.6 | 0.001 | 43.8 | 0.7 | 0.001 | 60.9 | 0.7 | 0.001 | 5.2 | 0.2 | 0.001 | 6.4 | 0.2 | 0.010 |
|  | CS_16 W vs CS_2 W | 17.4 | 0.4 | 0.001 | 24.4 | 0.5 | 0.001 | 17.7 | 0.4 | 0.001 | 18.8 | 0.5 | 0.001 | 3.7 | 0.1 | 0.001 | 12.7 | 0.4 | 0.002 |
|  | CS_16 W vs CS_4 W | 9.7 | 0.3 | 0.001 | 11.6 | 0.3 | 0.001 | 7.7 | 0.3 | 0.001 | 9.8 | 0.3 | 0.001 | 3.1 | 0.1 | 0.001 | 2.4 | 0.1 | 0.130 |
|  | CS_16 W vs CS_8 W | 19.9 | 0.5 | 0.001 | 14.6 | 0.4 | 0.001 | 16.3 | 0.4 | 0.001 | 15.3 | 0.4 | 0.001 | 5.3 | 0.2 | 0.001 | 2.8 | 0.1 | 0.069 |
|  | CS_2 W vs CS_4 W | 5.2 | 0.2 | 0.001 | 7.0 | 0.2 | 0.001 | 6.8 | 0.2 | 0.001 | 9.1 | 0.3 | 0.001 | 2.2 | 0.1 | 0.002 | 6.0 | 0.2 | 0.013 |
|  | CS_2 W vs CS_8 W | 14.0 | 0.4 | 0.001 | 16.7 | 0.4 | 0.001 | 19.9 | 0.5 | 0.001 | 39.0 | 0.6 | 0.001 | 5.3 | 0.2 | 0.001 | 14.5 | 0.4 | 0.001 |
|  | CS_4 W vs CS_8 W | 7.0 | 0.2 | 0.001 | 8.3 | 0.3 | 0.001 | 5.5 | 0.2 | 0.001 | 15.7 | 0.4 | 0.001 | 5.4 | 0.2 | 0.001 | 3.3 | 0.1 | 0.061 |
| YT | Global test | 13.4 | 0.5 | 0.001 | 28.9 | 0.7 | 0.001 | 14.6 | 0.5 | 0.001 | 38.5 | 0.7 | 0.001 | 16.6 | 0.5 | 0.001 | 8.6 | 0.4 | 0.001 |
|  | YT_1 W vs YT_16 W | 34.8 | 0.6 | 0.001 | 55.5 | 0.7 | 0.001 | 55.5 | 0.7 | 0.001 | 80.9 | 0.8 | 0.001 | 24.2 | 0.5 | 0.001 | 16.2 | 0.4 | 0.001 |
|  | YT_1 W vs YT_2 W | 3.0 | 0.1 | 0.024 | 14.5 | 0.4 | 0.001 | 14.5 | 0.4 | 0.001 | 19.7 | 0.5 | 0.001 | 1.6 | 0.1 | 0.024 | 4.0 | 0.2 | 0.019 |
|  | YT_1 W vs YT_4 W | 4.1 | 0.2 | 0.003 | 26.9 | 0.6 | 0.001 | 26.9 | 0.6 | 0.001 | 35.6 | 0.6 | 0.001 | 14.6 | 0.4 | 0.001 | 3.1 | 0.1 | 0.054 |
|  | YT_1 W vs YT_8 W | 16.9 | 0.4 | 0.001 | 40.7 | 0.6 | 0.001 | 40.7 | 0.6 | 0.001 | 115.3 | 0.8 | 0.001 | 23.0 | 0.5 | 0.001 | 10.6 | 0.3 | 0.002 |
|  | YT_16 W vs YT_2 W | 23.2 | 0.5 | 0.001 | 48.3 | 0.7 | 0.001 | 48.3 | 0.7 | 0.001 | 40.9 | 0.7 | 0.001 | 26.1 | 0.5 | 0.001 | 3.9 | 0.2 | 0.012 |
|  | YT_16 W vs YT_4 W | 16.4 | 0.4 | 0.001 | 25.6 | 0.5 | 0.001 | 25.6 | 0.5 | 0.001 | 19.2 | 0.5 | 0.001 | 19.7 | 0.5 | 0.001 | 3.9 | 0.2 | 0.062 |
|  | YT_16 W vs YT_8 W | 15.8 | 0.4 | 0.001 | 34.4 | 0.6 | 0.001 | 34.4 | 0.6 | 0.001 | 71.3 | 0.8 | 0.001 | 15.8 | 0.4 | 0.001 | 30.2 | 0.6 | 0.001 |
|  | YT_2 W vs YT_4 W | 4.3 | 0.2 | 0.009 | 11.1 | 0.3 | 0.001 | 11.1 | 0.3 | 0.001 | 11.3 | 0.3 | 0.001 | 13.0 | 0.4 | 0.001 | 1.8 | 0.1 | 0.134 |
|  | YT_2 W vs YT_8 W | 13.3 | 0.4 | 0.001 | 28.2 | 0.6 | 0.001 | 28.2 | 0.6 | 0.001 | 41.2 | 0.7 | 0.001 | 24.4 | 0.5 | 0.001 | 11.8 | 0.3 | 0.001 |
|  | YT_4 W vs YT_8 W | 7.8 | 0.3 | 0.001 | 13.7 | 0.4 | 0.001 | 13.7 | 0.4 | 0.001 | 33.2 | 0.6 | 0.001 | 10.5 | 0.3 | 0.001 | 14.0 | 0.4 | 0.001 |

**Table S3.** Mantel test between soil chemical properties and soil and straw-associated bacterial community composition across three experimental sites.

| **Soil chemical properties** | **Soil bacterial community composition** | | **Straw-associated bacterial community composition** | |
| --- | --- | --- | --- | --- |
|  | ***r*** | ***p*** | ***r*** | ***p*** |
| SOM | 0.797 | 0.001 | 0.515 | 0.001 |
| Total N | 0.509 | 0.001 | 0.335 | 0.001 |
| Total P | 0.870 | 0.001 | 0.563 | 0.001 |
| Total K | 0.923 | 0.001 | 0.621 | 0.001 |
| Available N | 0.430 | 0.001 | 0.283 | 0.001 |
| Available P | 0.943 | 0.001 | 0.623 | 0.001 |
| Available K | 0.826 | 0.001 | 0.540 | 0.001 |
| pH | 0.925 | 0.001 | 0.601 | 0.001 |

**Table S4.** Mantel test between straw chemical components and the dominant bacterial taxonomic and functional composition at local and regional scales.

| **Phylum** | **Straw chemistry** | **Microbial community** | | | | | | | | **Microbial function** | | | | | | | | |
| --- | --- | --- | --- | --- | --- | --- | --- | --- | --- | --- | --- | --- | --- | --- | --- | --- | --- | --- |
|  |  | **Local scale** | | | | | | **Regional scale** | | **Local scale** | | | | | | **Regional scale** | | |
|  |  | **CQ** | | **CS** | | **YT** | | **Across sites** | | **CQ** | | **CS** | | **YT** | | **Across sites** | | |
|  |  | ***r*** | ***p*** | ***r*** | ***p*** | ***r*** | ***p*** | ***r*** | ***p*** | ***r*** | ***p*** | ***r*** | ***p*** | ***r*** | ***p*** | ***r*** | ***p*** |  |
| *Actinobacteria* | All factors combined | **0.458** | **0.001** | **0.372** | **0.001** | **0.495** | **0.001** | **0.195** | **0.001** | **0.246** | **0.001** | **0.196** | **0.001** | **0.144** | **0.002** | **0.120** | **0.001** |  |
|  | Cellulose | 0.446 | 0.001 | 0.393 | 0.001 | 0.466 | 0.001 | 0.185 | 0.001 | 0.243 | 0.001 | 0.185 | 0.001 | 0.147 | 0.001 | 0.040 | 0.064 |  |
|  | Lignin | 0.467 | 0.001 | 0.439 | 0.001 | 0.481 | 0.001 | 0.268 | 0.001 | 0.270 | 0.001 | 0.221 | 0.001 | 0.084 | 0.016 | -0.049 | 0.965 |  |
|  | Hemicellulose | 0.329 | 0.001 | 0.210 | 0.001 | 0.510 | 0.001 | 0.151 | 0.001 | 0.042 | 0.205 | 0.104 | 0.013 | 0.093 | 0.008 | 0.018 | 0.215 |  |
|  | WSP | 0.208 | 0.002 | 0.327 | 0.001 | 0.304 | 0.001 | 0.299 | 0.001 | -0.107 | 0.986 | 0.179 | 0.001 | 0.046 | 0.216 | -0.113 | 1.000 |  |
| *Proteobacteria* | All factors combined | **0.610** | **0.001** | **0.563** | **0.001** | **0.625** | **0.001** | **0.355** | **0.001** | **0.312** | **0.001** | **0.418** | **0.001** | **0.384** | **0.001** | **0.258** | **0.001** |  |
|  | Cellulose | 0.580 | 0.001 | 0.541 | 0.001 | 0.571 | 0.001 | 0.357 | 0.001 | 0.298 | 0.001 | 0.355 | 0.001 | 0.374 | 0.001 | 0.203 | 0.001 |  |
|  | Lignin | 0.662 | 0.001 | 0.560 | 0.001 | 0.685 | 0.001 | 0.365 | 0.001 | 0.372 | 0.001 | 0.294 | 0.001 | 0.377 | 0.001 | 0.163 | 0.001 |  |
|  | Hemicellulose | 0.467 | 0.001 | 0.393 | 0.001 | 0.718 | 0.001 | 0.310 | 0.001 | 0.152 | 0.006 | 0.398 | 0.001 | 0.358 | 0.001 | 0.093 | 0.006 |  |
|  | WSP | 0.246 | 0.001 | 0.485 | 0.001 | 0.531 | 0.001 | 0.270 | 0.001 | -0.040 | 0.715 | 0.380 | 0.001 | 0.228 | 0.001 | -0.006 | 0.528 |  |
| *Firmicutes* | All factors combined | **0.556** | **0.001** | **0.420** | **0.001** | **0.458** | **0.001** | **0.187** | **0.001** | **0.319** | **0.001** | **0.329** | **0.001** | **0.172** | **0.001** | **0.163** | **0.001** |  |
|  | Cellulose | 0.535 | 0.001 | 0.359 | 0.001 | 0.413 | 0.001 | 0.220 | 0.001 | 0.292 | 0.001 | 0.165 | 0.009 | 0.126 | 0.009 | 0.134 | 0.001 |  |
|  | Lignin | 0.538 | 0.001 | 0.394 | 0.001 | 0.517 | 0.001 | 0.246 | 0.001 | 0.360 | 0.001 | 0.187 | 0.001 | 0.126 | 0.003 | 0.210 | 0.001 |  |
|  | Hemicellulose | 0.347 | 0.001 | 0.254 | 0.001 | 0.496 | 0.001 | 0.154 | 0.001 | 0.246 | 0.001 | 0.139 | 0.009 | 0.103 | 0.010 | 0.068 | 0.011 |  |
|  | WSP | 0.097 | 0.056 | 0.332 | 0.001 | 0.313 | 0.001 | 0.213 | 0.001 | 0.044 | 0.241 | 0.179 | 0.001 | 0.035 | 0.260 | 0.176 | 0.001 |  |
| *Bacteroidetes* | All factors combined | **0.594** | **0.001** | **0.615** | **0.001** | **0.396** | **0.001** | **0.362** | **0.001** | **0.157** | **0.001** | **0.438** | **0.001** | **0.216** | **0.001** | **0.221** | **0.001** |  |
|  | Cellulose | 0.480 | 0.001 | 0.585 | 0.001 | 0.413 | 0.001 | 0.319 | 0.001 | 0.151 | 0.001 | 0.411 | 0.001 | 0.216 | 0.001 | 0.213 | 0.001 |  |
|  | Lignin | 0.566 | 0.001 | 0.645 | 0.001 | 0.529 | 0.001 | 0.339 | 0.001 | 0.161 | 0.001 | 0.381 | 0.001 | 0.257 | 0.001 | 0.203 | 0.001 |  |
|  | Hemicellulose | 0.408 | 0.001 | 0.319 | 0.001 | 0.584 | 0.001 | 0.295 | 0.001 | 0.011 | 0.375 | 0.262 | 0.001 | 0.391 | 0.001 | 0.175 | 0.001 |  |
|  | WSP | 0.308 | 0.001 | 0.515 | 0.001 | 0.378 | 0.001 | 0.273 | 0.001 | -0.078 | 0.941 | 0.341 | 0.001 | 0.227 | 0.001 | 0.157 | 0.001 |  |
| *Acidobacteria* | All factors combined | **0.353** | **0.001** | **0.235** | **0.001** | **0.749** | **0.001** | **0.122** | **0.001** | **0.525** | **0.001** | **0.420** | **0.001** | **0.731** | **0.001** | **0.086** | **0.002** |  |
|  | Cellulose | 0.463 | 0.001 | 0.270 | 0.001 | 0.681 | 0.001 | 0.212 | 0.001 | 0.530 | 0.001 | 0.411 | 0.001 | 0.681 | 0.001 | 0.040 | 0.055 |  |
|  | Lignin | 0.431 | 0.001 | 0.292 | 0.001 | 0.776 | 0.001 | 0.240 | 0.001 | 0.505 | 0.001 | 0.455 | 0.001 | 0.830 | 0.001 | -0.049 | 0.972 |  |
|  | Hemicellulose | 0.251 | 0.001 | 0.257 | 0.001 | 0.742 | 0.001 | 0.206 | 0.001 | 0.338 | 0.001 | 0.340 | 0.001 | 0.787 | 0.001 | 0.018 | 0.201 |  |
|  | WSP | 0.030 | 0.314 | 0.307 | 0.001 | 0.627 | 0.001 | 0.179 | 0.001 | 0.137 | 0.015 | 0.476 | 0.001 | 0.614 | 0.001 | -0.113 | 1.000 |  |
| Bacteria | All factors combined | **0.671** | **0.001** | **0.550** | **0.001** | **0.633** | **0.001** | **0.323** | **0.001** | **0.442** | **0.001** | **0.525** | **0.001** | **0.531** | **0.001** | **0.205** | **0.001** |  |
|  | Cellulose | 0.619 | 0.001 | 0.533 | 0.001 | 0.566 | 0.001 | 0.343 | 0.001 | 0.449 | 0.001 | 0.490 | 0.001 | 0.503 | 0.001 | 0.186 | 0.001 |  |
|  | Lignin | 0.685 | 0.001 | 0.577 | 0.001 | 0.683 | 0.001 | 0.369 | 0.001 | 0.519 | 0.001 | 0.463 | 0.001 | 0.613 | 0.001 | 0.143 | 0.001 |  |
|  | Hemicellulose | 0.476 | 0.001 | 0.333 | 0.001 | 0.713 | 0.001 | 0.295 | 0.001 | 0.250 | 0.001 | 0.472 | 0.001 | 0.614 | 0.001 | 0.112 | 0.001 |  |
|  | WSP | 0.265 | 0.001 | 0.470 | 0.001 | 0.495 | 0.001 | 0.306 | 0.001 | -0.011 | 0.545 | 0.510 | 0.001 | 0.477 | 0.001 | 0.036 | 0.166 |  |

“All factors combined” were calculated using 4 chemical components of straw samples (Table S11) based on Euclidean distances

**Table S5.** Topological properties of networks of straw decomposition bacterial communities at each experimental site.

| **Network Indexes** | **CQ** | **CS** | **YT** |
| --- | --- | --- | --- |
| Total nodes | 215 | 180 | 130 |
| Total links | 572 | 547 | 336 |
| R square of power-law | 0.889 | 0.839 | 0.737 |
| Average degree (avgK) | 5.321 | 6.078 | 5.169 |
| Average clustering coefficient (avgCC) | 0.212 | 0.201 | 0.295 |
| Average path distance (GD) | 3.969 | 4.618 | 3.766 |
| Geodesic efficiency (E) | 0.313 | 0.296 | 0.325 |
| Harmonic geodesic distance (HD) | 3.191 | 3.376 | 3.075 |
| Maximal degree | 50 | 33 | 25 |
| Centralization of degree (CD) | 0.211 | 0.152 | 0.156 |
| Maximal betweenness | 3846 | 5173 | 2905 |
| Centralization of betweenness (CB) | 0.159 | 0.309 | 0.337 |
| Maximal stress centrality | 86703 | 43626 | 42346 |
| Centralization of stress centrality (CS) | 3.579 | 2.557 | 4.891 |
| Maximal eigenvector centrality | 0.289 | 0.259 | 0.323 |
| Centralization of eigenvector centrality (CE) | 0.253 | 0.22 | 0.283 |
| Density (D) | 0.025 | 0.034 | 0.04 |
| Transitivity (Trans) | 0.085 | 0.213 | 0.353 |
| Connectedness (Con) | 0.769 | 0.861 | 0.811 |
| Efficiency | 0.973 | 0.967 | 0.959 |
| Modularity | 0.465 | 0.537 | 0.578 |

**Table S8.** Description of three field experimental sites ([Wu et al., 2019](#_ENREF_3)).

| **Site** | **CQ** | **CS** | **YT** |
| --- | --- | --- | --- |
| Province | Chongqing | Jiangsu | Jiangxi |
| Longitude | 106°25' | 120°42' | 116°55' |
| Latitude | 29°49' | 31°33' | 28°15' |
| Altitude (m) | 200 | 3 | 20 |
| Climate | subtropical monsoon | subtropical monsoon | subtropical monsoon |
| MAP (mm) | 1106 | 1321 | 1795 |
| MAT (°C） | 18.4 | 16.6 | 17.6 |
| Fertilization  (ha^-1^ yr^-1^) | 285 kg N  120 kg P_2_O_5_  120 kg K_2_O | 360 kg N  150 kg P_2_O_5_  300 kg K_2_O | 230 kg N  136 kg P_2_O_5_  84 kg K_2_O |
| Straw  (ha^-1^ yr^-1^) | 7500 kg | 4500 kg | 4500 kg |
| Soil classification | grayish brown purple soil developed from Shaximiao Formation | Gleyic-Stagnic Anthrosols (WRB-FAO) developed from lake sediment | red paddy soil derived from Quaternary red clay |
| Soil texture | 27.0% sand, 51.7% silt and 21.3% clay | 13.3% sand, 54.8% silt and 31.9% clay | 20.6% sand, 41.0% silt and 38.4% clay |

MAP, mean annual precipitation; MAT, mean annual temperature.

**Table S9.** Soil chemical properties of three experimental sites ([Wu et al., 2019](#_ENREF_3)).

| **Site** | **SOM** | **Total N** | **Total P** | **Total K** | **Available N** | **Available P** | **Available K** | **pH** |
| --- | --- | --- | --- | --- | --- | --- | --- | --- |
|  | **g kg^-1^** | **g kg^-1^** | **mg kg^-1^** | **g kg^-1^** | **mg kg^-1^** | **mg kg^-1^** | **mg kg^-1^** | **1:2.5** |
| CQ | 27.2±2.1 | 1.4 ±0.1 | 775 ±18 | 25.4 ±0.6 | 94 ±2.7 | 33.2 ±1.0 | 106 ±4 | 7.0 ±0.1 |
| CS | 36.4 ±2.3 | 2.1 ±0.1 | 870 ±19 | 22.3 ±0.6 | 134 ±8.1 | 24.8 ±2.1 | 141 ±6 | 7.2 ±0.3 |
| YT | 18.4 ±2.2 | 1.1 ±0.1 | 534 ±57 | 15.7 ±0.7 | 84 ±9.1 | 2.6 ±0.4 | 70 ±7 | 5.2 ±0.1 |

Data are means of 3 replicates with standard deviation.

Soil organic matter (SOM) was determined using a volumetric K_2_Cr_2_O_7_-heating method. Soil total N was determined by Kjeldahl digestion. Soil total P and K were first digested by hydrofluoric acid (HF)-perchloric acid (HClO_4_) and then determined by molybdenum-blue colorimetry and flame photometry, respectively. Available N content was measured using the alkaline hydrolysis method. Available P in the soil was extracted by sodium bicarbonate and determined using the molybdenum-blue method. Available K in the soil was extracted by ammonium acetate and determined by flame photometry. Soil pH was determined from soil-water suspensions (1:2.5 v/v).

**Table S10.** Straw decomposition ratios over 16-week decomposition stages ([Bao et al., 2020a](#_ENREF_1)).

| **Time (week)** | **CQ** | **CS** | **YT** |
| --- | --- | --- | --- |
| 1 | 25.63±3.57a | 26.85±4.47a | 13.08±3.80a |
| 2 | 30.85±3.92b | 31.83±2.37b | 18.08±4.05a |
| 4 | 37.67±3.66c | 37.95±1.81c | 29.46±5.01b |
| 8 | 49.75±7.49d | 51.25±3.24d | 40.36±2.48c |
| 16 | 65.75±5.82e | 55.5±2.79e | 48.28±6.70d |

Different letters denotes significant differences (*P* < 0.05).

**Table S11.** Concentration of straw chemical components over 16-week decomposition stages ([Bao et al., 2020b](#_ENREF_2)).

| **Site** | **Time (Week)** | **Cellulose (%)** | **Lignin**  **(%)** | **Hemicellulose (%)** | **WSP**  **(%)** |
| --- | --- | --- | --- | --- | --- |
| CQ | 1 | 34.52±5.30e | 9.50±0.44e | 11.24±1.33c | 0.29±0.01bc |
|  | 2 | 31.16±2.55d | 7.86±0.93d | 10.99±0.90c | 0.31±0.05c |
|  | 4 | 21.06±3.16c | 6.14±0.48c | 7.99±0.56b | 0.29±0.02bc |
|  | 8 | 14.51±1.38b | 5.26±0.68b | 8.04±1.58b | 0.27±0.03b |
|  | 16 | 8.74±1.36a | 3.18±0.38a | 4.61±1.24a | 0.20±0.05a |
| CS | 1 | 30.53±3.34d | 7.14±0.39d | 12.40±1.48c | 0.25±0.02d |
|  | 2 | 30.18±1.98d | 6.94±0.26d | 12.11±2.25c | 0.26±0.02d |
|  | 4 | 25.58±1.77c | 6.07±0.45c | 8.97±1.02b | 0.22±0.02c |
|  | 8 | 19.26±1.00b | 4.14±0.31b | 9.06±0.93b | 0.19±0.01b |
|  | 16 | 14.33±2.57a | 3.45±0.12a | 6.53±0.86a | 0.17±0.01a |
| YT | 1 | 39.22±7.16d | 13.39±0.77e | 14.32±0.82e | 0.48±0.05d |
|  | 2 | 42.63±6.08d | 12.33±0.26d | 13.62±0.64d | 0.42±0.06c |
|  | 4 | 28.30±2.21c | 8.38±0.66c | 10.49±0.85c | 0.33±0.01b |
|  | 8 | 18.24±1.28b | 6.19±0.58b | 8.42±0.32b | 0.31±0.02b |
|  | 16 | 14.36±1.98a | 5.22±0.72a | 6.70±0.77a | 0.26±0.04a |

Different letters denotes significant differences (*P* < 0.05).


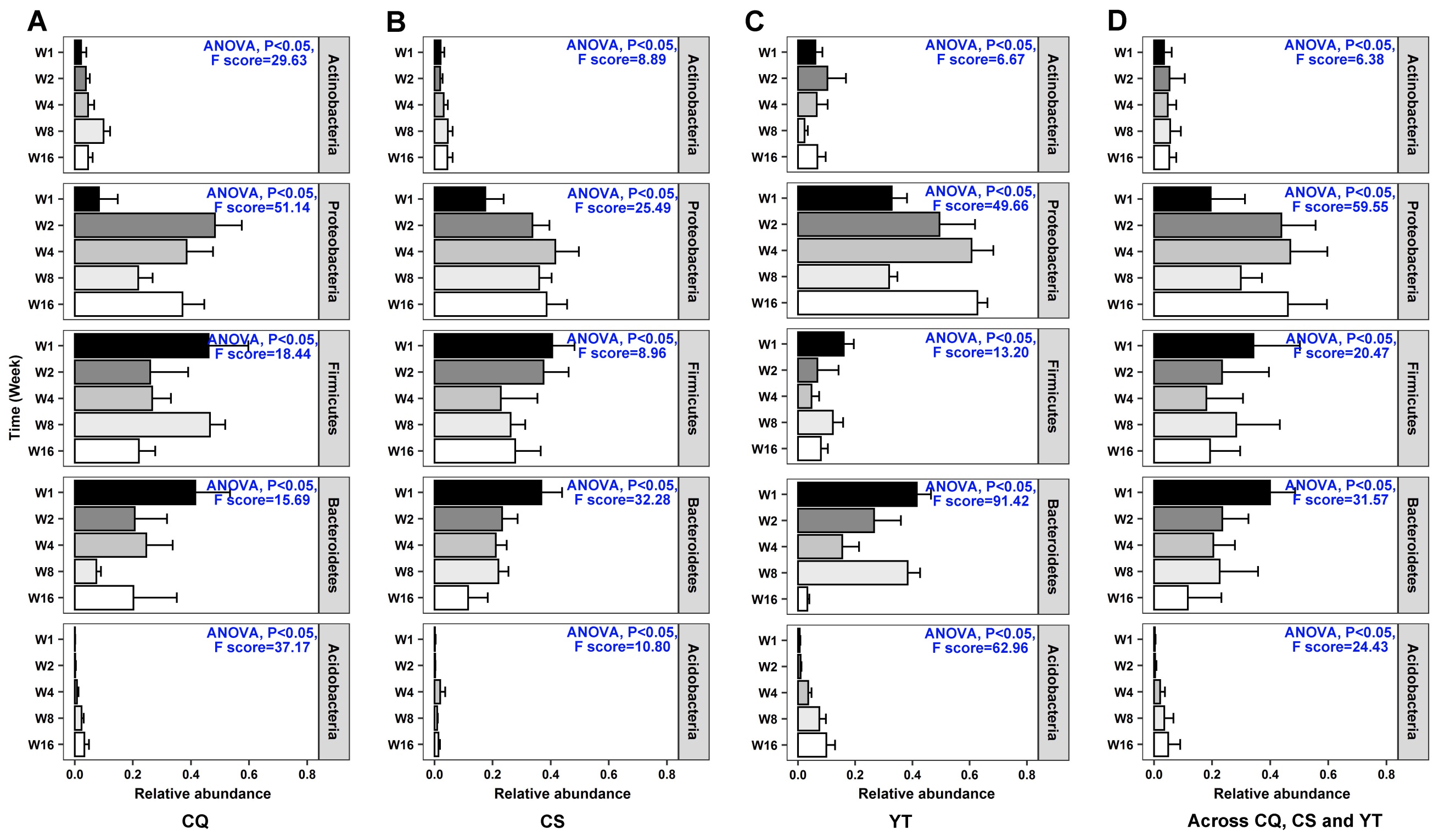


**Figure S1.** Relative abundances of dominant bacteria across decomposition stages at the phylum level in CQ (A), CS (B), YT (C), and across three experimental sites (D).


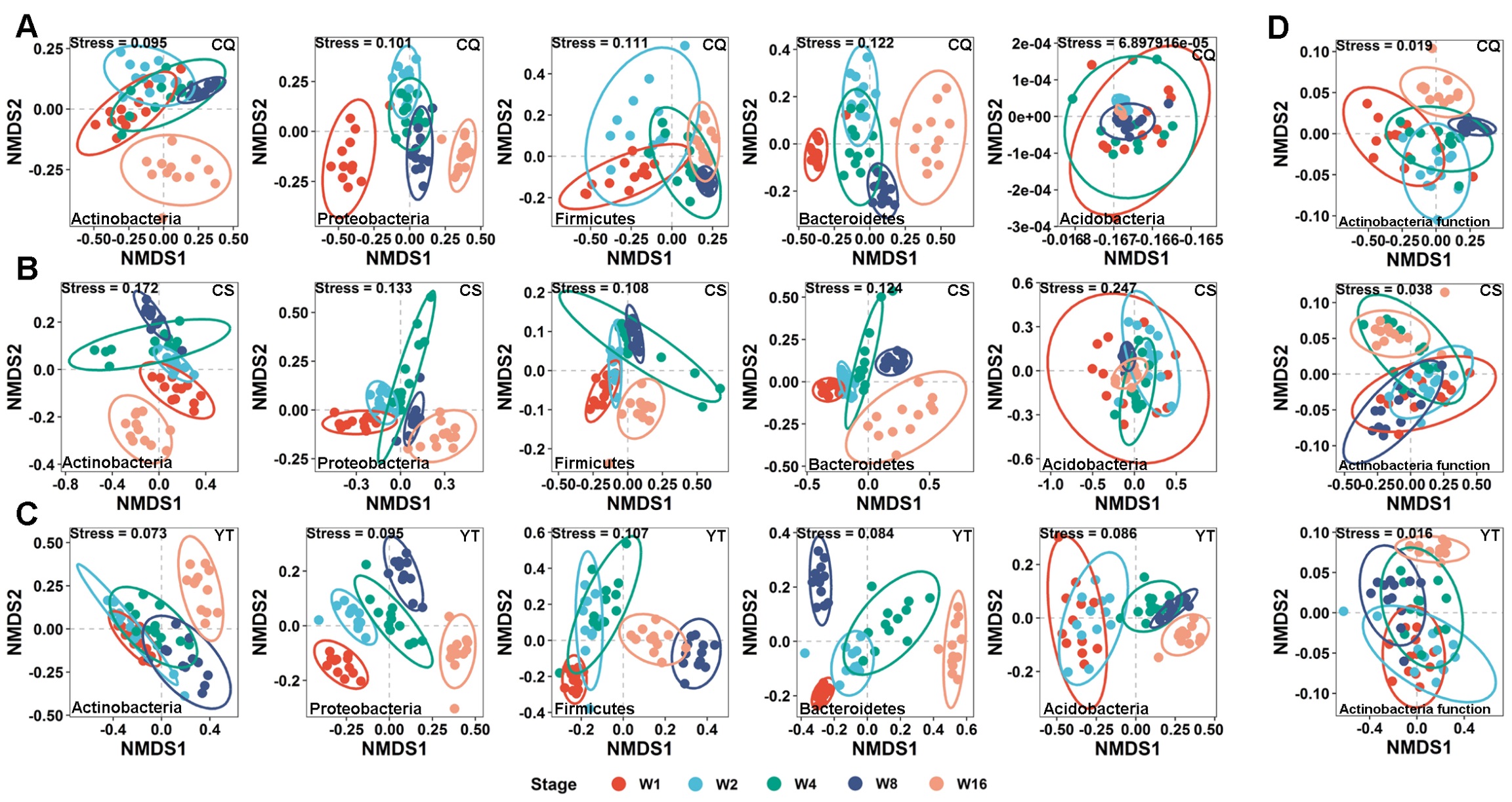


**Figure S2.** Nonmetric multidimensional (NMDS) analysis of the dominant straw-associated bacterial taxonomic (A-C) and *Actinobacteria* functional (D) composition between different decomposition stages, based on Bray-Curtis distance, in CQ, CS, and YT (n = 60, each plot). The circles indicate a 95% standard error of each stage.


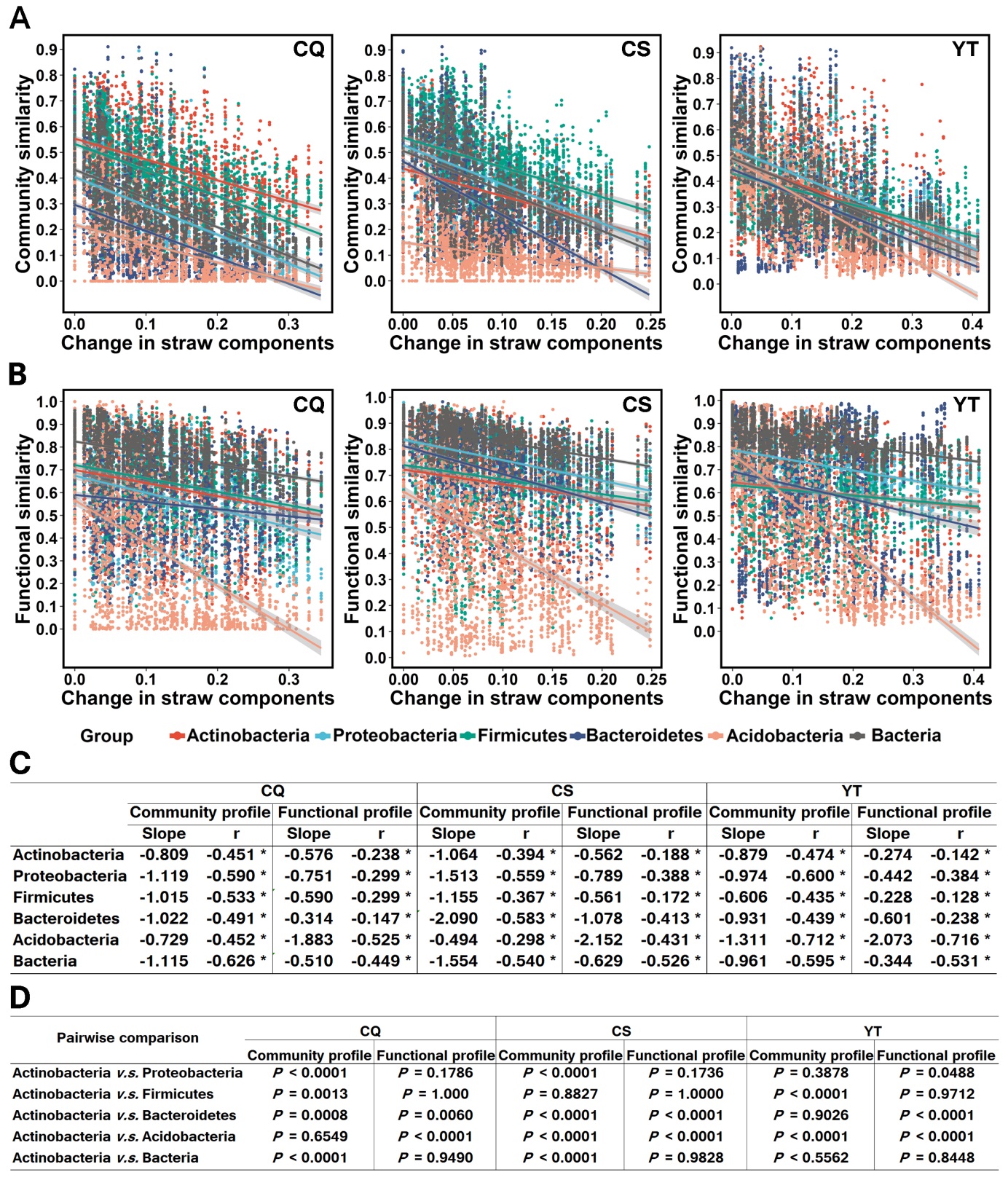


**Figure S3.** Distance matrix regressions between straw chemistry and community (A) and functional (B) composition during decomposition within each experimental site. The slops and correlation coefficients of linear models are provided (C) as well as the significance of the linear regression slopes between *Actinobacteria* and other members tested by permutation tests (D). Horizontal axes indicate Euclidean distances based on all straw components. “*” denotes that the slopes were significantly less than zero by permutation tests at *P* < 0.0001.


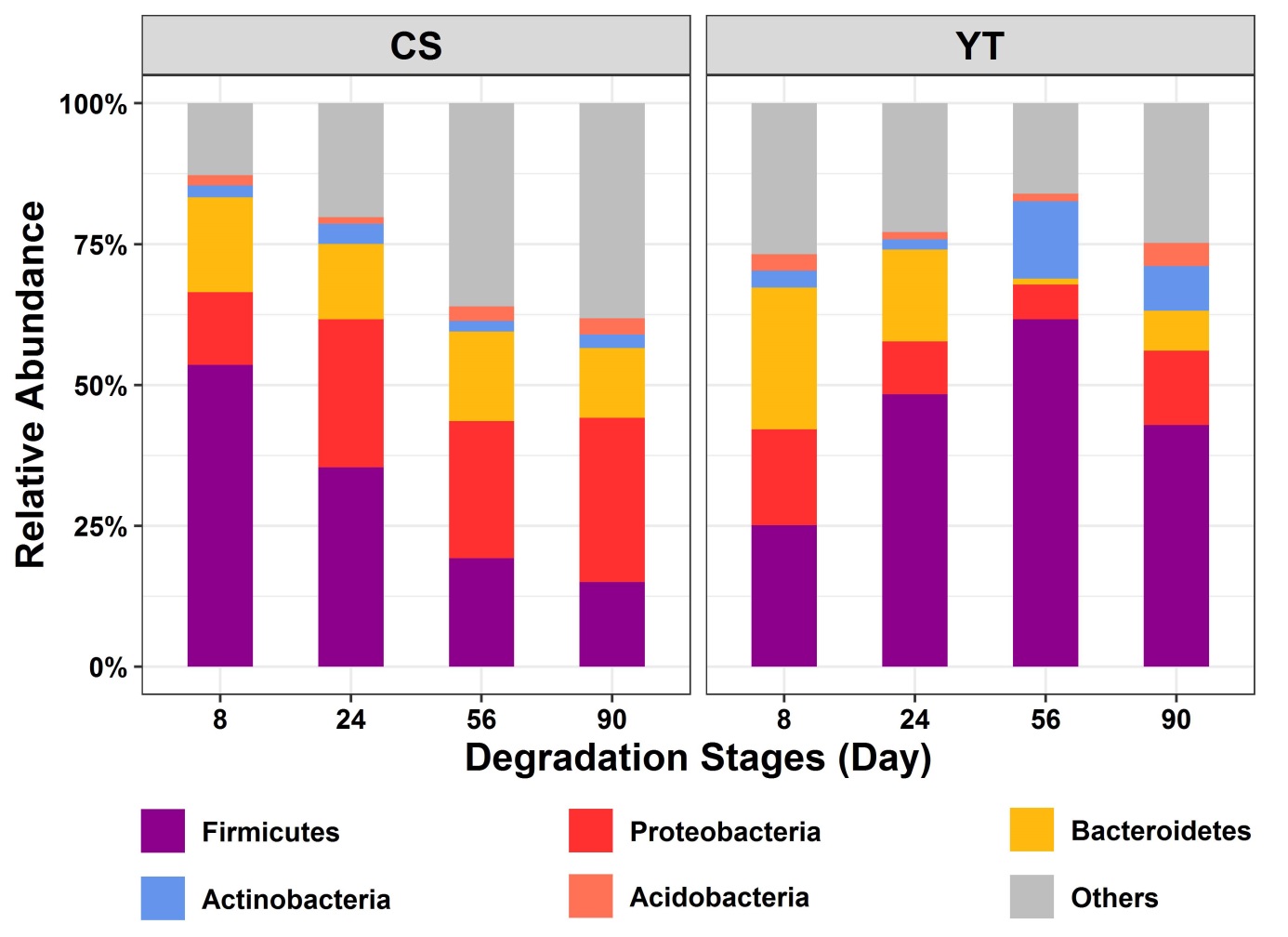


**Figure S4.** Relative abundances of dominant bacteria at the phylum level revealed by DNA-SIP based shotgun metagenomic sequencing.


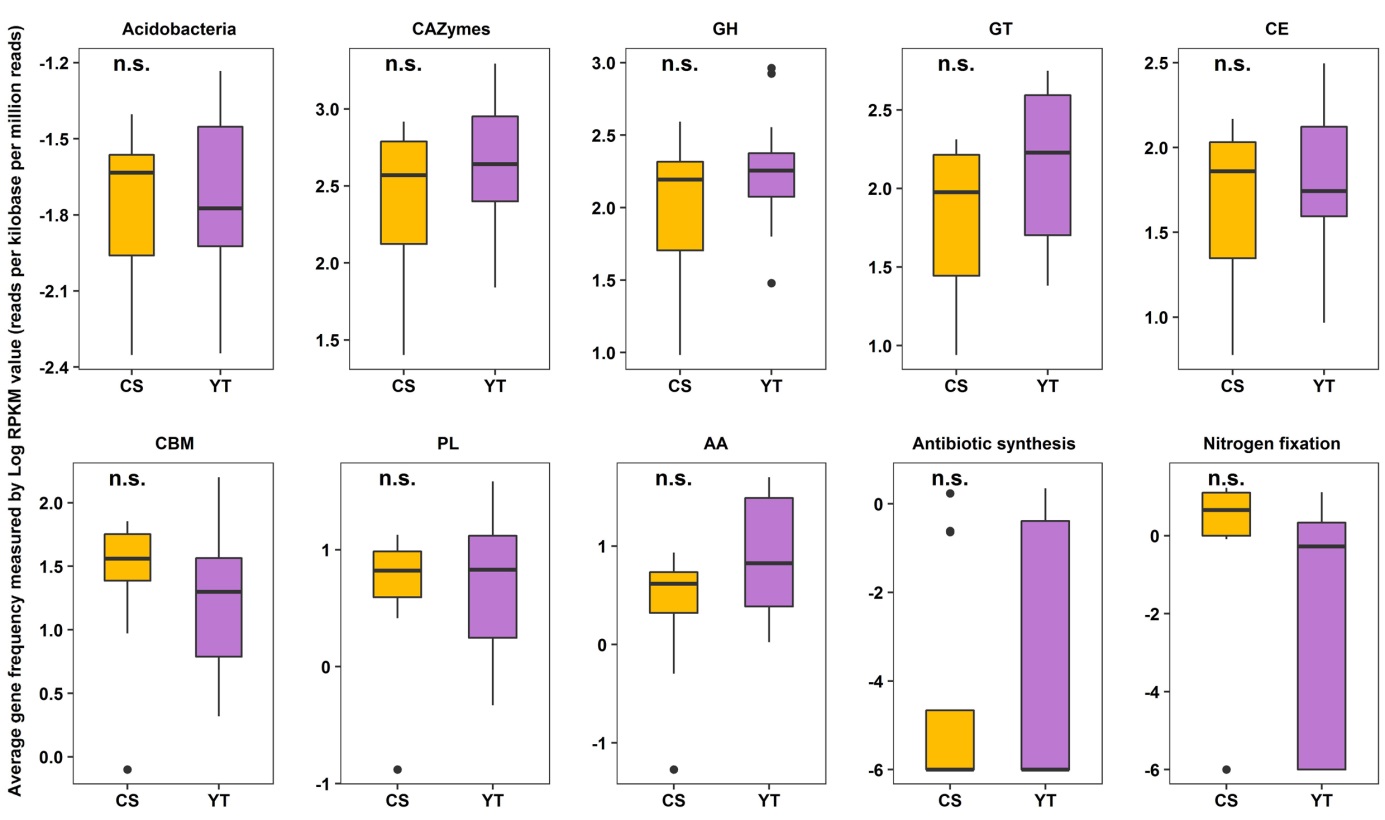


**Figure S5.** Boxplots showing the average relative abundance of *Acidobacteria* and related gene classes revealed by DNA-SIP based shotgun metagenomic sequencing that associated with different ecological traits in *Acidobacteria* metagenome under different soil fertility. The relative abundances were log-transformed. GH: Glycoside hydrolase, GT: glycosyl transferase, PL: polysaccharide lyase, CE: carbohydrate esterase, CBM: carbohydrate-binding module, and AA: auxiliary activities. “n.s.” denotes *P* > 0.05.


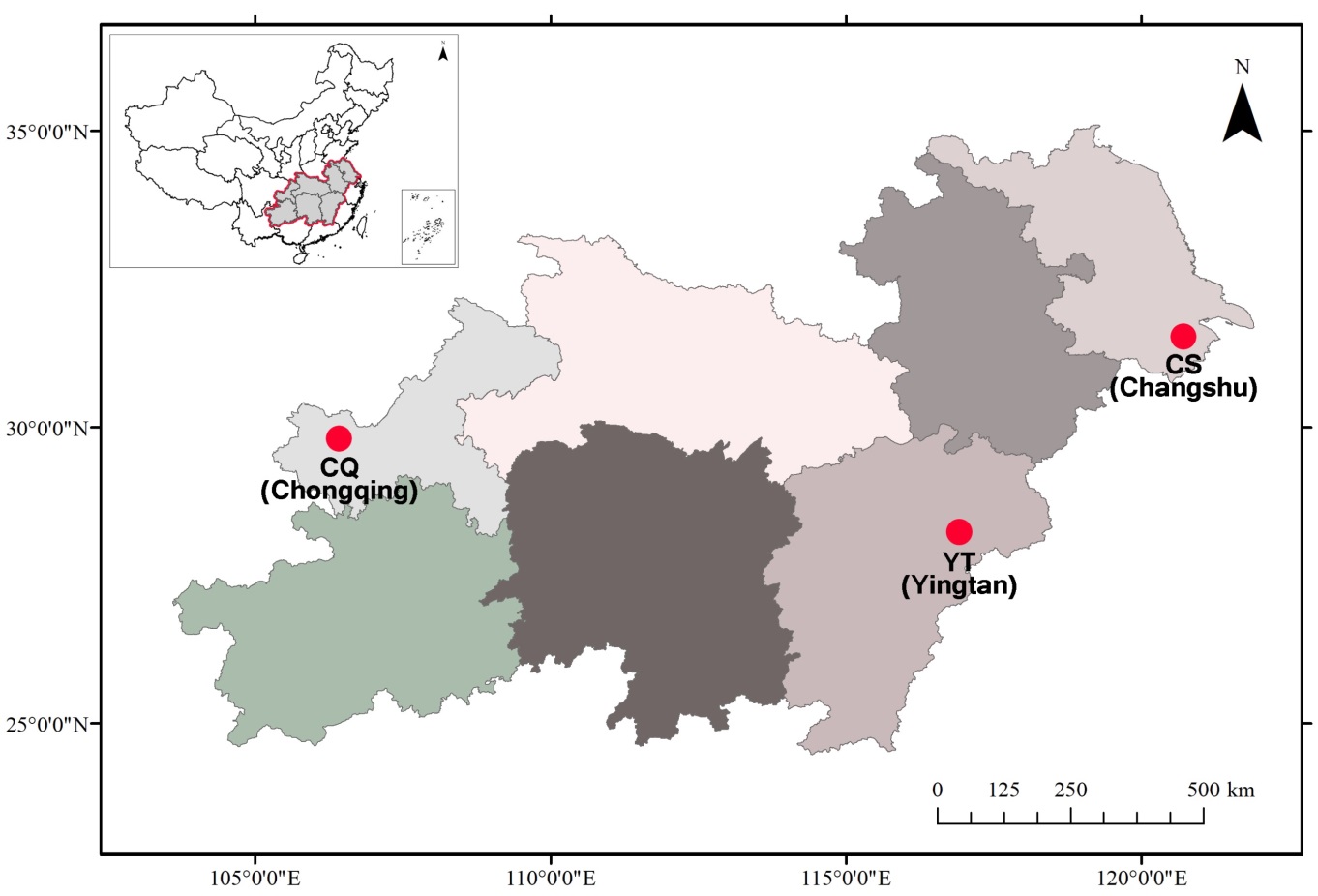


**Figure S6.** Locations of the three field experimental sites (CQ, CS, and YT).

**
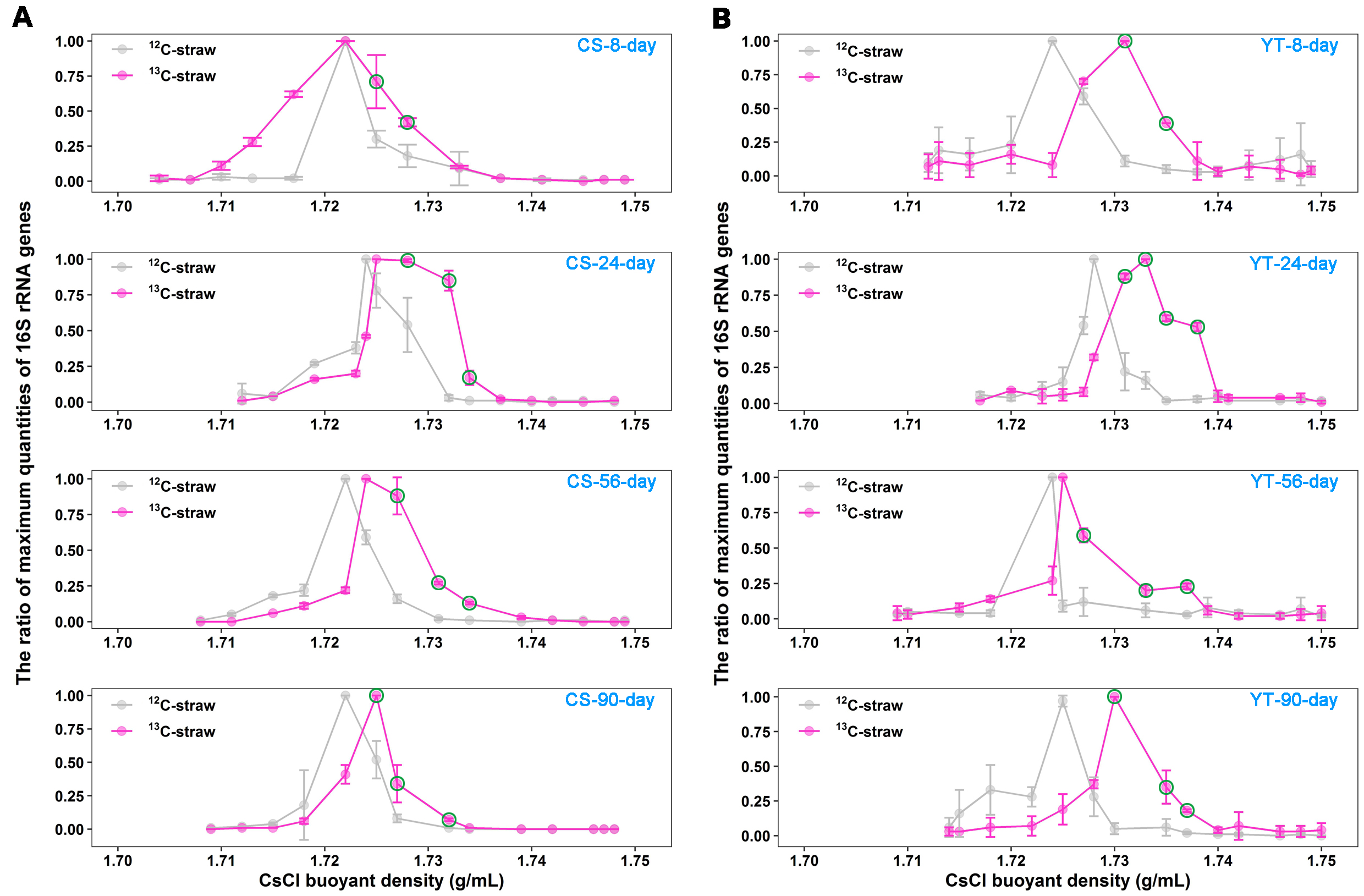
**

**Figure S7.** Distributions of the copy numbers of bacterial 16S rRNA gene across the buoyant densities of the DNA gradients isolated from soil samples treated with ^13^C- or with ^12^C-straw in CS (A) and YT (B). DNA fractions of ^13^C-straw treatments with green circles were defined as “heavy” genomic DNA fractions and were then subjected to shotgun metagenomic sequencing.

**Reference:**

Bao, Y.Y., Feng, Y.Z., Stegen, J.C., Wu, M., Chen, R.R., Liu, W.J., Zhang, J.W., Li, Z.P., Lin, X.G., 2020a. Straw chemistry links the assembly of bacterial communities to decomposition in paddy soils. Soil Biology and Biochemistry, 107866.

Bao, Y.Y., Guo, Z.Y., Chen, R.R., Wu, M., Li, Z.P., Lin, X.G., Feng, Y.Z., 2020b. Functional community composition has less environmental variability than taxonomic composition in straw-degrading bacteria. Biology and Fertility of Soils 56, 869-874.

Wu, M., Zhang, J.W., Bao, Y.Y., Liu, M., Jiang, C.Y., Feng, Y.Z., Li, Z.P., 2019. Long-term fertilization decreases chemical composition variation of soil humic substance across geographic distances in subtropical China. Soil and Tillage Research 186, 105-111.

Zheng, W., Zhao, Z., Gong, Q., Zhai, B., Li, Z., 2018. Effects of cover crop in an apple orchard on microbial community composition, networks, and potential genes involved with degradation of crop residues in soil. Biology and Fertility of Soils 54, 743-759.
